# Computational screening of piezoelectric constants in metal–organic frameworks: design principles and ferroelectric-like bond modulation

**DOI:** 10.1039/d5ta09332e

**Published:** 2026-02-24

**Authors:** Srinidhi Mula, Chunyu Huang, Ferdinand Grozema, Monique A. van der Veen

**Affiliations:** a Department of Chemical Engineering, Technische Universiteit Delft Delft 2629HZ The Netherlands m.a.vanderveen@tudelft.nl

## Abstract

Piezoelectric energy harvesting is a process in which energy in the form of kinetic movements can be harvested and converted into useful electrical energy using piezoelectric materials. Metal–organic frameworks (MOFs) have a huge potential for piezoelectric energy harvesting owing to their high flexibility, structural tunability, and very low dielectric constants due to their high porosity. The piezoelectric constant *d* relevant for piezoelectric energy harvesting depends on the piezoelectric constant *e* and the flexibility of the structure (*i.e.* mechanical properties). The mechanical properties of MOFs have previously been extensively studied but the piezoelectric constant *e* was never explored for MOFs. In this work, we generate a database of piezoelectric properties, specifically *e* for around ∼1608 previously synthesized non-centrosymmetric MOF structures. The calculations were performed using the density functional perturbation theory (DFPT) method. The highest piezoelectric constant *e* obtained in this work is approximately ∼2.76 C m^−2^, which is significantly higher than that of the flexible organic piezoelectric polymer polyvinylidene fluoride (PVDF) and its copolymers. In this work, we analyze and identify structural factors that influence the values of the piezoelectric constant for high-performing MOFs. Based on that, a series of guidelines for the design of MOF structures that can lead to a high piezoelectric constant *e* are presented. One class of high-performing piezoelectric MOFs is based on polar patterns of O—(short)—Mo—(long)—O unequal bond length, reminiscent of ferroelectric inorganic oxides. This class could have potential for ferroelectricity, meaning that the bond length pattern could be reversed by external electrical field. We substantiate this by showing experimentally *via* SHG-microscopy that the O—(short)—Mo—(long)—O unequal bond lengths are indeed malleable by external conditions.

## Abstract 2

This study computes the piezoeletric tensor *e* of metal–organic frameworks (MOFs) using DFPT, analyzes structure–property relationships for high-*e* MOFs, and evaluates their mechanical properties and piezoelectric constants *d*.

## Graphical abstract

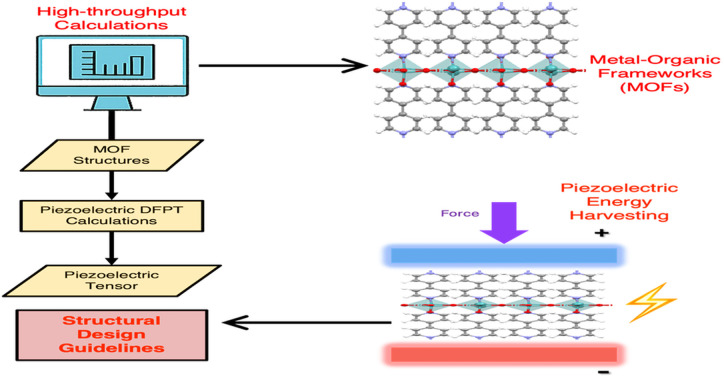

## 1 Introduction

A major application of piezoelectric materials that is of wide interest to researchers is piezoelectric energy harvesting. This technology has large potential for self-powering in environmental monitoring, asset tracking, portable technologies, and remote “Internet of Things” (IoT) nodes and sensors, *via* conversion of ambient mechanical movement to electricity through the piezoelectric effect.^1–4^ The phenomenon of piezoelectricity describes the coupling between the mechanical and electrical properties of materials. This is observed in non-centrosymmetric crystalline structures, where either an electric dipole moment is generated on application of mechanical stress or when mechanical strain is induced on the application of the electric field.^5^ The performance of an energy harvester in practical applications is improved by a higher piezoelectric constant *e* (unit [C m^−2^]), a higher mechanical compliance *s* (unit [m^2^ N^−1^]) and a lower dielectric constant or relative permittivity *ε* (unitless). Note that the piezoelectric constant *d* (unit [pC N^−1^]) equals *d* = *es*.^6,7^

Existing piezoelectrics that have been explored for energy-harvesting applications include conventional materials like inorganic ceramics, organic polymers, and composites of organic–inorganic materials. Inorganic ceramic oxides of ABO_3_ type (PZT-PbZrTiO_3_, BaTiO_3_, LiNbO_3_, *etc.*) have very high piezoelectric constants *d* ranging from 69 pC N^−1^ to 410 pC N^−1^.^8,9^ However, they are brittle, have high densities and require high processing temperatures, making them unfavorable for wearable and flexible device applications. Several techniques have been used by researchers to fabricate them into thin films and nanowires to form relatively flexible systems compared to bulk materials. However, these materials generally have high dielectric constants (25–1700), thus reducing the efficiency of energy harvesting.^8,9^ On the other hand, organic polymers are naturally flexible, durable, and easy to process compared to inorganic piezolectrics. The most common piezoelectric polymers include poly(vinylidene fluoride) (PVDF), its copolymers poly(vinylidene fluoride-*co*-trifluoroethylene) (PVDF-TrFE). Although the piezoelectric response of polymers is low (1.5 pC N^−1^ to 50 pC N^−1^) compared to inorganic piezoelectrics, they have a much lower dielectric constant (12); thereby enhancing the figure of merit (FoM) for energy harvesting efficiency.^9,10^ In the recent years, hybrid inorganic-organic perovskites and metal-free perovskites emerged as potential candidates for piezoelectric energy harvesting. Organic–inorganic hybrid perovskites have a general formula of ABX_3_ (A = organic anion, B = metal ions, and X = halogens, CN^−^, NO^−^_2_, *etc.*) and metal-free organic perovskites with general formula A(NH_4_)X_3_. They can be processes by solution-based methods and show a high piezoelectric response (*d*) that is on par with that of conventional inorganic ceramics.^11–13^ The piezoelectric constant values *d* of hybrid perovskites range from 185 pC N^−1^ to 240 pC N^−1^ while for metal-free perovskites they range from 178 pC N^−1^ to 248 pC N^−1^.^11–15^ Both hybrid and metal-free perovskites offer great chemical diversity and structural tuneability because of their hybrid nature and organic components. However, the dielectric constant of these perovskites is around 35, not as low as the organic polymers. Existing piezoelectric materials have both advantages and limitations in intrinsic properties for energy harvesting. Therefore, there is a need to explore novel materials with improved intrinsic material properties to maximize the figure of merit (FoM) and improve piezoelectric energy harvesting efficiency.

Metal–organic frameworks (MOFs) are porous hybrid crystalline materials consisting of inorganic nodes connected to organic ligands *via* coordination bonds. By properly selecting metal nodes and organic linkers in MOFs, the structure can be tuned to achieve the desired properties for the target application. The mechanical properties of MOFs and the intrinsic relationship between structural changes such as linker effects, interpenetration on mechanical properties have been previously discussed in the literature.^16–18^ MOFs exhibit anisotropic mechanical properties, which means that their strength and stiffness vary depending on the direction of force applied. This anisotropy is primarily due to the underlying structure of the MOF, where different directions are dominated by rigid inorganic chains or flexible organic ligands.^19,20^ This is an influencing factor for the piezoelectric tensorial properties *d* which depends on the magnitude of the piezoelectric constant *e* and the elastic compliance *s* in a specific direction. The elastic compliance constant tensor *s* relevant for piezoelectric constant *d* has not been reported for many MOFs, but regularly the Young's and Shear moduli of MOFs are reported in the literature. MOFs show a low shear modulus (1.0 GPa to 40.8 GPa) compared to Young's modulus (3 GPa to 142 GPa) suggesting that they are more prone to deformation under shear stress than uniaxial stresses.^21–25^ The low values for Young's moduli and shear moduli for MOFs compared to inorganic piezoelectrics such as LiNbO_3_, PbTiO_3_, BaTiO_3_ (29 GPa to 251 GPa)^26,27^ indicate the high flexibility of MOFs; making them favorable for applications in flexible devices. Furthermore, MOFs have an inherent permanent porosity in the structure, leading to very low dielectric constants (2.3 to 6.0).^28–36^ MOFs due to their flexible frameworks (expected large *s*), tuneability and low dielectric constants show great potential as piezoelectric materials.

The piezoelectric response *d* in MOFs was measured experimentally by quasi-static measurements (measured using a PM200 piezometer) in two MOFs: in one a *d*_33_ value of 60.10 pC N^−1^ was measured for MOFs based on Cd[imazethapyr]^37^ in the other, the *d*_22_ value of Mn/Co-based homochiral coordination framework Mn_2_(D − cam)_2_(2-Hpao)_4_ where (D − cam = D − (^+^) − camphoricacid; 2-Hpao = 2-pyridinealdoxime) is measured to be 6.9 pC N^−1^.^33^ In another work, the piezoelectric tensor was theoretically computed for three MOFs; QMOF-1 (Zn(ISN)_2_; ISN = isonicotinate), QMOF-2 (InH(BDC)_2_; BDC = 1,4-benzendicarboxylate), and [DMA][Mg(HCOO)_3_] (DMA = (CH_3_)_2_(NH_2_)^+^). The values of the components of tensor *d*_ip_ for these MOFs range from 2 pC N^−1^ to 23 pC N^−1^.^38^ The MOFs whose piezoresponse was studied in these works differ widely in their building units and structure. Hence, it is difficult to derive the structure–property relationships of piezoelectric properties that will guide the design of better-performing piezoelectric MOFs. In our previous work, we explored in detail the structure–property relationships of piezoelectric constants *e* and *d*, and thus the compliance *s* for Zeolitic Imidazolate Frameworks (ZIFs).^39^ The metal node M (Zn or Cd) and the substituent on the imidazolate linker in the structure of ZIFs were varied and the piezoelectric constants were calculated for a set of six ZIFs. The computed piezoelectric constants *d* and the estimated figure of merit (FoM) for piezoelectric energy harvesting for CdIF-1 (Cd metal node and – CH_3_ substituent in imidazolate linker) show high potential as efficient energy harvesters. Specifically in CdIF-1, high flexibility of the framework is key to obtain a high piezoelectric constant *d* (∼46 pC N^−1^) that is comparable to *d* of widely known organic piezoelectric P(VDF-TrFE) (∼50 pC N^−1^). In the case of ZIFs, the *e* values for all structures were low (<0.01 C m^−2^). Hence, tuning the structural building units of MOFs not just for high mechanical flexibility (high *s*), but also to obtain high piezoelectric constants *e* should lead to even further enhanced values of *d*, and thus to high performance in mechanical energy harvesting. As discussed above, the mechanical properties of MOFs have been widely studied, but the piezoelectric constant *e* remains largely unexplored. Hence, here, we focus on unraveling the structure–property relationships for *e* of MOFs.

In this work, we computed the piezoelectric constant *e* for a large dataset of experimentally synthesized and computationally ready MOF structures using Density Functional Perturbation Theory (DFPT).^40–42^ Similar high-throughput calculations of the piezoelectric constant *e* and mechanical properties were performed previously for inorganic crystalline materials^26,43^ and the computational results were made publicly available. Such databases can enable the accelerated discovery of new materials with novel mechanical and piezoelectric properties. The goal of this work is to compute the piezoelectric constants *e* and analyze them to identify the trends and factors that can influence the values *e* of these structures. The influence of the structure symmetry and Born effective charges of the inorganic cations in the secondary building units of the MOFs on the piezoelectric tensor of the MOFs is presented. Finally, for the top MOF candidates with values *e* higher than 1.0 C m^−2^, we discuss the structural features that are responsible for such high values. Based on that, we provide a series of guidelines for the design of MOF structures that can contribute to a high piezoelectric constant *e*. Among the MOF candidates with high *e*, the piezoelectric constant *d* was calculated for four MOFs. The values of the piezoelectric constant (*e*, *d*) and the elastic compliance constant (*s*) for these four MOFs are compared with the values of some traditional inorganic and organic piezoelectrics.

## 2 Theory

The response of a piezoelectric material to mechanical stress/strain and electric fields is mathematically described in the IEEE standard for piezoelectricity.^44^ The piezoelectric constants that are of importance in piezoelectric energy harvesting are *d* and *e* with units [pC N^−1^] and [C m^−2^] respectively. The piezoelectric constant *d* represents the coupling between mechanical stress and electric polarization in a material and, conversely, how an applied electric field results in a deformation (strain) in the material. The piezoelectric constant *e* describes the coupling between mechanical deformation (strain) and electric polarization in the material. The piezoelectric tensors *d*_ip_ and *e*_iq_ are related through the mechanical properties of the material, specifically the elastic compliance tensor *s*_qp_ as given by *d*_ip_ = *e*_iq_*s*_qp_. The elastic compliance constant *s* with units [m^2^ N^−1^] is an indication of the flexibility of the material. Detailed theory of piezoelectricity, voigt notation for tensors, and computational methodology of *e*_iq_ and *d*_ip_ are described in the SI Section 1.

The piezoelectric tensor *e*_iq_ can be decomposed into two distinct contributions: the clamped-ion (or frozen-ion) and the internal strain (relaxed-ion) contributions (see eqn (1)).^45^ The clamped-ion contribution (*e*^0^_iq_) is computed by applying strain to the unit cell while keeping the fractional atomic coordinates fixed. The Cartesian coordinates change because of the deformation of the lattice vectors. This results in an electronic response (*P*_i_), which captures the redistribution of electron density under strain (*S*_q_) without ionic relaxation.

The internal strain contribution (*e*^int^_iq_), on the other hand, includes the part of the electronic response (*P*_i_) when the atoms are allowed to relax in response to the applied strain (*S*_q_). This allows the atoms to shift from their strained fractional positions to new equilibrium positions, giving rise to additional polarization due to ionic displacements. The Born effective charges (Z*, BEC) and the relative changes in the positions of atoms (*x*_sp_) during relaxation are included in the internal strain contribution. The Born effective charge (BEC), introduced by Max Born and Maria Göppert-Mayer, describes the change in polarization induced by an atomic displacement under a zero macroscopic electric field. In high-performance piezoelectric perovskites of the ABO_3_ type, the anomalously large BECs of the transition-metal (M) cations contribute directly to their enhanced piezoelectric constants *e*. These pronounced BECs originate from the mixed covalent–ionic character of the M–O bonds. Dynamical changes in orbital hybridization upon displacement lead to significant charge redistribution which is observed as the BEC of atoms.

The total piezoelectric tensor is expressed as the sum of these components:1
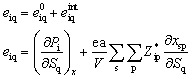


The performance of piezoelectric energy harvesters is described by its figure of merit (FoM) defined as

2 FoM = *d*^2^/*ε*_0_*ε*^*T*^ = (*es*)^2^/*ε*_0_*ε*^*T*^

With units of m^2^ N^−1^, where *ε*^*T*^ is the relative permittivity (or dielectric constant) of the material under constant stress (*T*). The FoM is commonly used to evaluate the suitability of materials for energy harvesting applications.^6,7^ A high FoM indicates a more efficient energy harvester, which can be achieved by optimizing intrinsic properties of materials such as a high piezoelectric response (*d*, *e*), mechanical properties (high elastic compliance constant *s*) and low dielectric constant *ε*.

## 3 Results and discussions

For the selection of MOF structures for piezoelectric calculations, we start from a computationally ready database of MOFs called the QMOF database (v13), consisting of 20425 structures.^46^ Various selection criteria such as non-centrosymmetric, experimentally synthesized MOF structures with atoms in the primitive cell less than 150 were applied to these MOF structures (summarized in detail in the SI in Section 2.1), leading to shortlisting of 1608 MOFs for which piezoelectric tensors were calculated in this work. The convergence of these 1608 piezoelectric calculations was further evaluated based on a few consistency checks (see SI Section 2.4 for details), thus leading to final piezoelectric tensors and piezoelectric constants for around 1263 MOFs, 79% of the number of initial MOF structures.

Note here that the piezoelectric tensor *e*_iq_ is a third rank tensor with 18 terms. For comparison between the piezoelectric properties of different MOF structures, we adopt a simplified value for *e*_iq_ that has previously been used in high-throughput calculations for inorganic piezoelectrics.^43^ The simplified value is the norm value of the final piezoelectric tensor and is denoted ‖*e*_iq_‖_max_. This value represents the maximum attainable absolute value of the longitudinal piezoelectric response measured along a specific crystallographic direction compared to all other possible directions.

### 3.1 Effect of non-polar and polar symmetry on the piezoelectric norm ‖*e*_iq_‖_max_

**Fig. 1:**
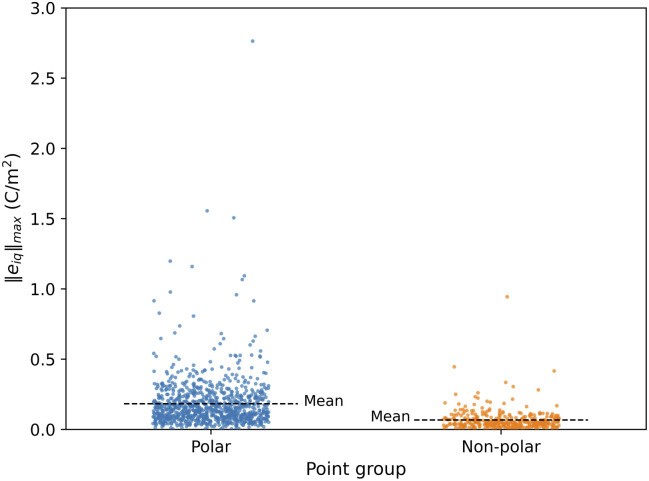
Categorical plot showing the distribution of ‖*e*_iq_‖_max_ values for polar and non-polar MOFs.

The ‖*e*_iq_‖_max_ values for MOFs in the final dataset range between 0.004 C m^−2^ and 2.76 C m^−2^. A structure is considered non-centrosymmetric if its space group lacks an inversion center, identified through its corresponding non-centrosymmetric point group. Among these, polar non-centrosymmetric structures have an inbuilt spontaneous polarization that exists even in the absence of external electric fields and external stress/strain. This is mainly due to the intrinsic asymmetric arrangement of atoms in the structure. In contrast, non-polar non-centrosymmetric structures do not have this inbuilt spontaneous polarization. In Fig. 1, we show the distribution of piezoelectric constant ‖*e*_iq_‖_max_ values for polar and non-polar MOF structures. For non-polar point group MOFs, the distribution's mean value is 0.1 C m^−2^ and for polar point groups it is 0.2 C m^−2^*i.e.* relatively similar. However, very high ‖*e*_iq_‖_max_ values greater than ∼0.5 C m^−2^ are observed mostly for polar MOFs and for one MOF with a non-polar point group. Hence, a high value of the piezoelectric constant *e* is more likely in structures that have spontaneous polarization. MOFs with ‖*e*_iq_‖_max_ greater than 0.5 C m^−2^ belong to polar point groups 1, 2, m, mm2, and 4.

In the case of inorganic piezoelectrics, the magnitude of the DFT calculated values ‖*e*_iq_‖_max_ for a large fraction of compounds is <1.0 C m^−2^.^43^ However, 17% of the compounds had values ≥1.0 C m^−2^ with a maximum value of 20 C m^−2^.^43^ This is almost an order of magnitude higher than the maximum value for MOFs (∼2.7 C m^−2^) in this work. Inorganic piezoelectrics with ‖*e*_iq_‖_max_ greater than 2.5 C m^−2^ belong to the polar point groups (1, 2, mm^2^, 3, 3 m, 6 mm, 4, and 4 mm), while non-polar point group (32, −6 m^2^, −42 m, and −43 m) inorganic piezoelectrics have ‖*e*_iq_‖_max_ values between 1.0 C m^−2^ to 2.5 C m^−2^. The piezoelectric response for polar structures is a combination of spontaneous polarization effects and strain-induced charge redistribution, typically yielding a higher response than is observed in non-polar structures where spontaneous polarization is absent. Hence, in case of both inorganic piezoelectrics and MOFs, polar point group structures show high values of piezoelectric constant *e* than non-polar point group structures.

Inorganic ceramics with superior piezoelectric properties such as PZT (PbZr_*x*_Ti_1−*x*_O_3_, tetragonal), BaTiO_3_ and SrTiO_3_ have *e*_iq_ values of 11.9 C m^−2^, 6.7 C m^−2^ and 9.3 C m^−2^, respectively, and organic polymers like PVDF, P(VDF-TrFE) have magnitudes for *e*_iq_ as 0.27 C m^−2^ and 0.18 C m^−2^.^10,47–49^ The metal-free organic piezoelectric MDABCO–NH_4_–X_3_ (with MDABCO: *N*-methyl-*N′*-diazabicyclo[2,2,2] octonium) has *e*_iq_ values in the range of 0.1 C m^−2^ to 0.3 C m^−2^.^12^ The piezoelectric constant values for MOFs in this work range from 0.004 C m^−2^ to 2.76 C m^−2^. Compared to traditional inorganic ceramics, MOFs have lower piezoelectric constant values *e*, whereas MOFs have higher *e* values compared to organic polymers and metal-free perovskites.

### 3.2 Born effective charge (BEC, Z*) of inorganic cations in MOFs

In high-performing inorganic piezoelectrics of ABO_3_ type, anomalously high Born effective charges (BEC's) of the metal atoms are responsible for the high piezoelectric constants *e*. The concept of BEC is defined and discussed in SI Section 1. For example, in BaTiO_3_, SrTiO_3_, and PbTiO_3_, Ti with an oxidation state (OS) of +4, has a BEC of +6.7 to +7.6, *i.e.*, BEC values of Ti range from (OS + 2.7) to (OS + 3.6). The *e* values for BaTiO_3_, PbTiO_3_ and SrTiO_3_ is 6.7, 3.23 and 9.3 C m^−2^.^47,50,51^ Motivated by these observations, we investigate the magnitude and trends in the BEC of the inorganic cations for MOFs in this work. The Born effective charge (BEC) is a second-rank tensor with nine terms. In most cases, the highest change in the BEC with a change in the position of the atoms is expected to occur in the 11, 22, and 33 directions. Hence, for the comparison of BECs of inorganic cations in different MOFs the average value of the diagonal elements (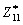, 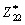, 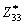) of the BEC tensor is used as a representative value for the BEC.

The distribution of BEC values for inorganic cations in MOFs categorized by their respective groups in the periodic table is shown in Fig. S5 in the SI. A detailed discussion of the variation in BEC values with the group of the periodic table is included in SI section 4. To summarize, the BEC values of most of the inorganic cations range from (OS) to (OS + 2). This indicates that, unlike inorganic ceramics, the born effective charge values of inorganic cations in MOFs are not anomalously high. BEC values of +4 and higher are observed among elements in groups 3, 4, 5, 6, 15 and the f block.

**Fig. 2:**
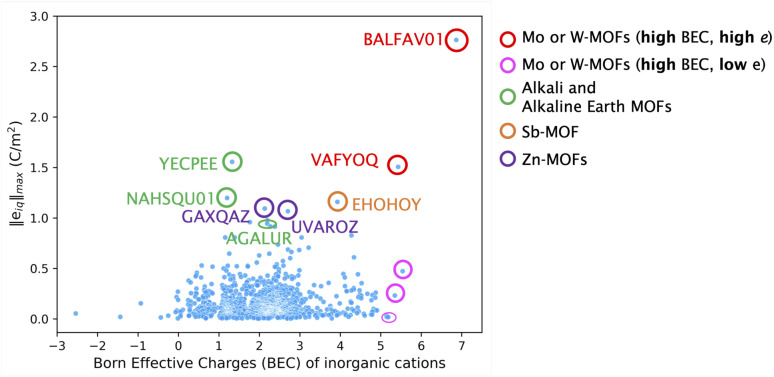
Born Effective Charge (BEC) values of inorganic cations in MOFs *versus* the piezoelectric norm ‖*e*_iq_‖_max_ of the MOF. The CSD refcode for the MOFs with high ‖*e*_iq_‖_max_ values are also indicated in the figure.

Fig. 2 illustrates the relationship between the BEC value of the inorganic cation and the value of norm piezoelectric constant (‖*e*_iq_‖_max_) of the corresponding MOF. For most MOFs, BEC values typically range from +1 to +3. However, certain inorganic cations can exhibit significantly higher BEC values, between +5.0 and +7.0, comparable to the value of +6.7 observed for Ti in BaTiO_3_. This is observed for the secondary building units with Mo and W elements of group 6 (circled in red and pink in Fig. 2). In fact, the BEC of Mo in BALFAV01 and VAFYOQ (red circles) in a particular direction 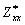 and 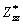 is almost +9.0 and +8.7 respectively. Unlike inorganics, where high BEC usually leads to high *e* it is different in MOFs. As highlighted in red and pink circles in Fig. 2, the BEC of inorganic cations in Mo and W-based MOFs is higher than +5, however, this does not necessarily lead to a higher *e* of the MOF. A high “*e*” can be obtained for both high and low values of born effective charge (BEC) of inorganic cations as evident from the top MOF candidates named in Fig. 2. So in contrast to inorganics, while high BEC values may contribute, they are not necessarily the reason for a high “*e*” in MOFs.

### 3.3 Clamped-ion (‖*e*^0^_iq_‖_max_) and internal strain (‖*e*^int^_iq_‖_max_) contributions to piezoelectric norm ‖*e*_iq_‖_max_

**Fig. 3:**
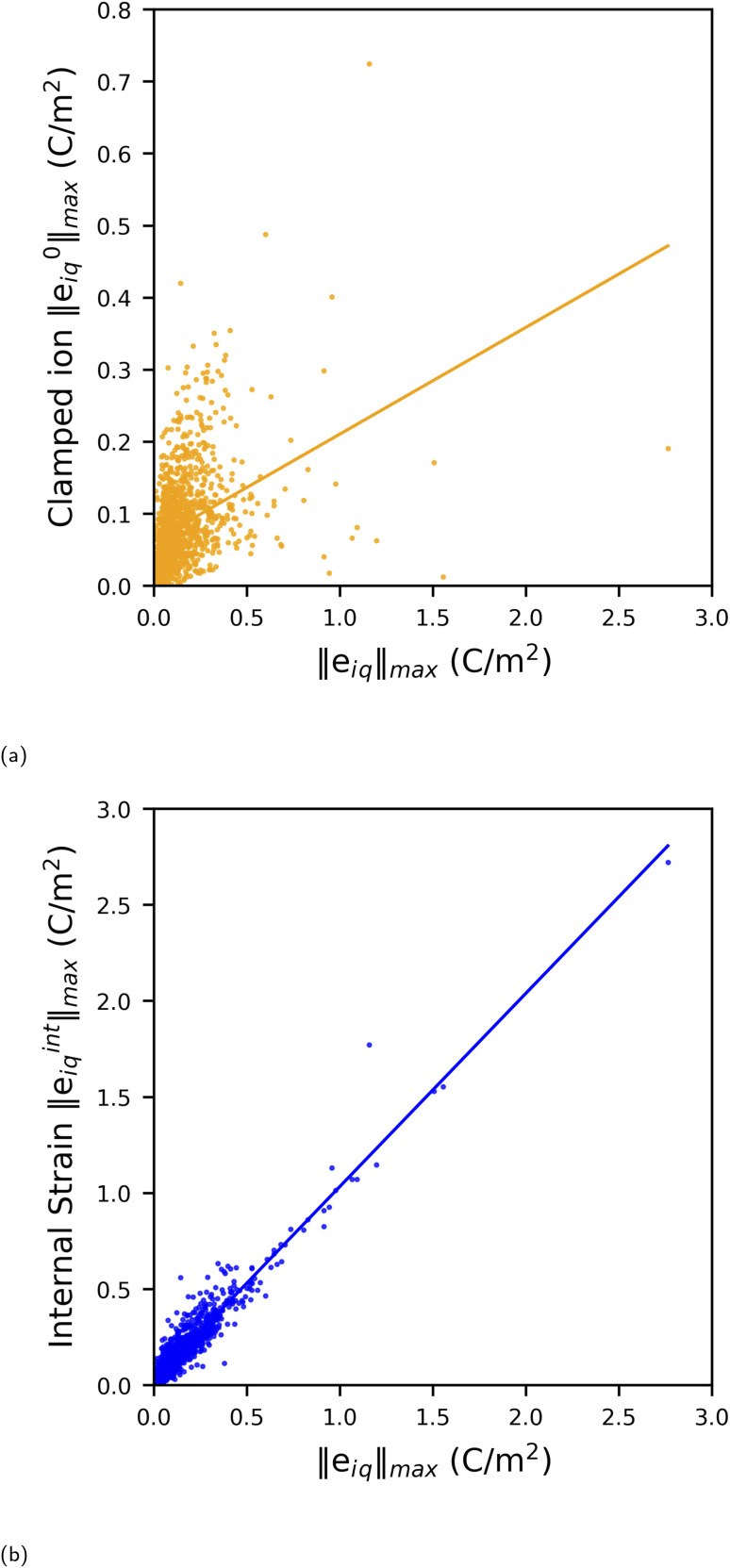
Correlation between (a) clamped ion ‖*e*^0^_iq_‖_max_ (b) internal strain ‖*e*^int^_iq_‖_max_ and total piezoelectric norm ‖*e*_iq_‖_max_ values.

As explained in theory in Section 2, the piezoelectric tensor *e*_iq_ can be divided into clamped-ion (*e*^0^_iq_) and internal strain (*e*^int^_iq_) contributions. To understand the relative contributions of the clamped-ion and internal strain terms to the piezoelectric tensor *e*, we compare the norm values of the clamped-ion tensors (‖*e*^0^_iq_‖_max_) and internal strain (‖*e*^int^_iq_‖_max_) tensors with the norm values of the piezoelectric tensor ‖*e*_iq_‖_max_ in Fig. 3a and b. In this work, the values of the total piezoelectric constant for MOFs range from 0.004 to 2.76 C m^−2^, while the clamped-ion values vary from 2 × 10^−5^ to 0.724 C m^−2^, and the internal strain contribution values vary from 0.001 to 2.721 C m^−2^.

A linear fit between clamped ion, internal strain and total piezoelectric norm is included in Fig. 3. Statistically, the Pearson correlation coefficient is used to measure the strength of linear correlation between two variables. The values can range from +1 to −1, where a positive value indicates that they tend to increase together; a negative value indicates that one value increases when the other value decreases. The closer the Pearson coefficient to +1 or −1, the stronger the relationship between the two variables. Here, the value of Pearson's correlation coefficient between the internal strain (‖*e*^int^_iq_‖_max_) and piezoelectric tensor norm ‖*e*_iq_‖_max_ is 0.95; whereas for clamped-ion (‖*e*^0^_iq_‖_max_) and piezoelectric tensor norm (‖*e*_iq_‖_max_) it is 0.37. Thus, the internal strain contribution ‖*e*^int^_iq_‖_max_ is strongly correlated to the piezoelectric tensor ‖*e*_iq_‖_max_ and is a dominant factor for obtaining a higher *e* value in MOFs. The internal strain contribution is due to the combined effect of the BEC of all atoms and the relative changes in the positions of atoms due to applied lattice strain. Hence, to understand these contributions structurally, in our discussion of MOFs with a high piezoelectric *e* norm (‖*e*_iq_‖_max_) we will elaborate on the structural factors that could lead to a higher ‖*e*^int^_iq_‖_max_ and thus ‖*e*_iq_‖_max_ value in these MOFs in the next section.

### 3.4 Detailed discussion of selected MOFs

**Fig. 4:**
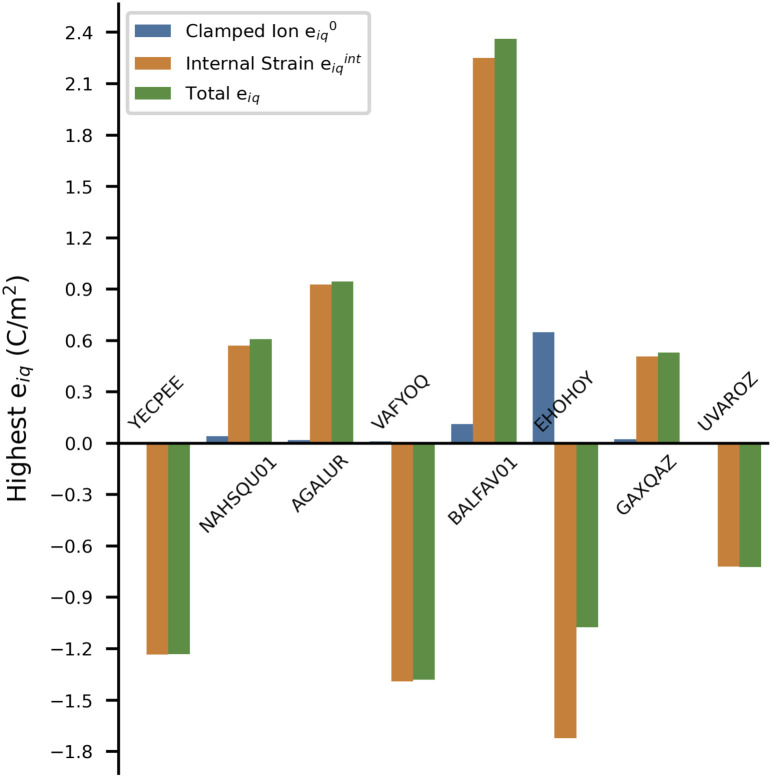
Bar plot of highest *e*_iq_ and the respective clamped ion and internal strain contributions in the same iq direction for the eight MOFs with the highest piezoelectric norm value ‖*e*_iq_‖_max_.

Here, we discuss all MOFs with ‖*e*_iq_‖_max_ values ≥1.0 C m^−2^ in detail. There are seven MOFs that have ‖*e*_iq_‖_max_ ≥ 1.0 C m^−2^, all of which belong to polar point groups. We also discuss the non-polar structure (AGALUR) with highest ‖*e*_iq_‖_max_ that is slightly lower than 1.0 C m^−2^. Fig. 4 shows a graph of the highest total values *e*_iq_, clamped ion values *e*^0^_iq_ and internal strain values *e*^int^_iq_ for these eight MOFs along with their reference code used in the CSD database. Note here that to compare the magnitude of clamped-ion and internal strain piezoelectric constants in the same direction, we show the highest *e*_iq_ value of the tensor (not the norm values, *i.e.*, ‖*e*_iq_‖_max_) and the clamped-ion and internal strain value in the same iq direction in the bar plot in Fig. 4. The complete piezoelectric tensor *e*_iq_ and the BEC of inorganic cations for these eight MOFs are included in the SI Section 6.

As observed in the bar plot (Fig. 4), the clamped-ion contribution *e*^0^_iq_ is quite low compared to the total *e*_iq_ value for each MOF, except for EHOHOY. In fact, EHOHOY has the highest clamped-ion *e* of the entire dataset. Clearly, for the eight MOFs, the internal strain value *e*^int^_iq_ dominates the high *e*_iq_ values.

#### 3.4.1 Alkali and alkaline earth metal based structures (YECPEE, NAHSQU01, and AGALUR)

**Fig. 5:**
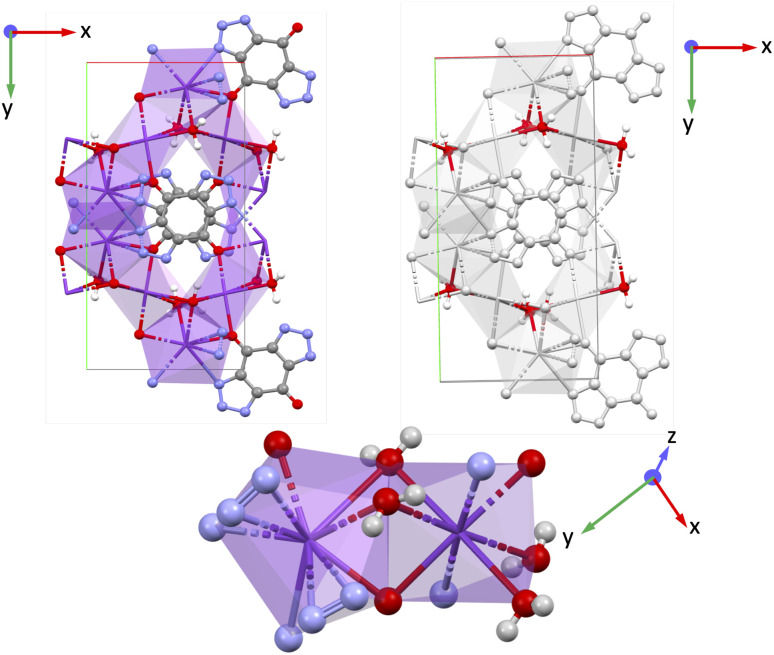
Structure of YECPEE shown along the *xy* plane, and the coordination environment around the inorganic cation K for YECPEE. Purple: potassium (K), gray: carbon (C), red: oxygen (O), white: hydrogen (H), blue: nitrogen (N).

**Fig. 6:**
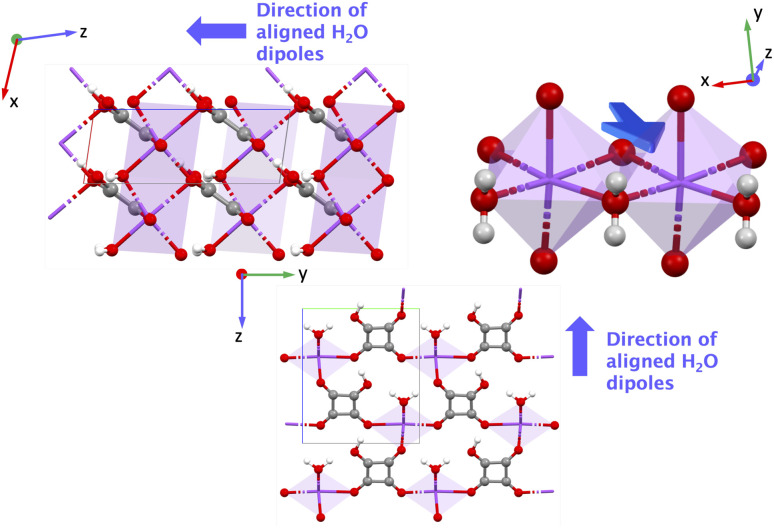
Structure of NAHSQU01 shown along the *xz*, *yz* planes, and the coordination environment around the inorganic cation Na for NAHSQU01. Purple: sodium (Na), gray: carbon (C), red: oxygen (O), white: hydrogen (H), blue: nitrogen (N).

YECPEE and NAHSQU01 based on alkali metal ions K^+^ and Na^+^ respectively belong to the polar monoclinic point group ‘*m*’ where all the non-zero values of the piezoelectric tensor *e*_iq_ involve the *x* and/or *z*-directions because the structure has inversion symmetry along the *y*-direction. YECPEE (K_2_- btbq·2H_2_O) contains bistriazole-*p*-benzoquinone (btbq), a centrosymmetric linker^52^ whereas in NAHSQU01 (NaHC_4_O_4_·H_2_O) hydrogensquarate is the linker, which is polar.^53^ Both MOFs have water molecules coordinated to inorganic cations as shown in Fig. 5 and 6. The total piezoelectric norm values ‖*e*_iq_‖_max_ for YECPEE and NAHSQU01 are 1.556 C m^−2^ and 1.198 C m^−2^. The low BEC values (Z*) of the inorganic cations (K: +1.36, Na: +1.20) do not explain the high *e* values for these MOFs. For both MOFs, the piezoelectric tensor components with the highest magnitude all involve a polarization observed along the *z*-direction, with a strain or shear applied along the *x*- and/or *z*-direction. For YECPEE, the highest value in the piezoelectric tensor is *e*_31_ = −1.233 C m^−2^ (the full piezoelectric tensor is shown in the SI in Section 6.1). Similarly, for NAHSQU01 *e*_33_ = 0.565 C m^−2^ and *e*_35_ = 0.607 C m^−2^ are very close in magnitude and are the highest values in the tensor (the full piezoelectric tensor is shown in the SI in Section 6.2).

For YECPEE, the non-centrosymmetric order that stands out when visualizing the *xy*-plane is the coordinated water molecules that are arranged in an overall polar manner along the *z*-direction (see highlighted in Fig. 5, top right). Additionally, we analyzed the dielectric tensor (specifically the relaxed-ion part) and observed that it is large along the *z*-direction, *ε*_*zz*_ = 15.471, compared to *x*- and *y*-directions (*ε*_*xx*_ = 2.892 and *ε*_*yy*_ = 4.607). The dielectric tensor represents the measure of the polarizability of the lattice structure, and in this case, indicates a large contribution from highly polarizable water molecules in the presence of an electric field along *z*. The complete clamped-ion and relaxed-ion dielectric tensor is shown in the SI Section 7.1. For NAHSQU01, the organization of the water molecules with their dipoles aligned along the *z*-direction and the orientation of the hydroxy group of the organic ligands along the *x* and *z*-directions stand out (Fig. 6). The dielectric tensor (ionic part) for NAHSQU01 is high along *xx*, *xz*, *zx*, and *zz*, confirming the large polarizability of hydroxy groups and water molecules in the structure. The complete clamped-ion and relaxed-ion dielectric tensor is shown in the SI Section 7.2. In other words, in both YECPEE and NAHSQU01, the water molecules (and for NAHSQU01 the hydroxy groups of the organic ligands) are polarly aligned along those directions and highly polarizable, thus contributing to high *e* values.

**Fig. 7:**
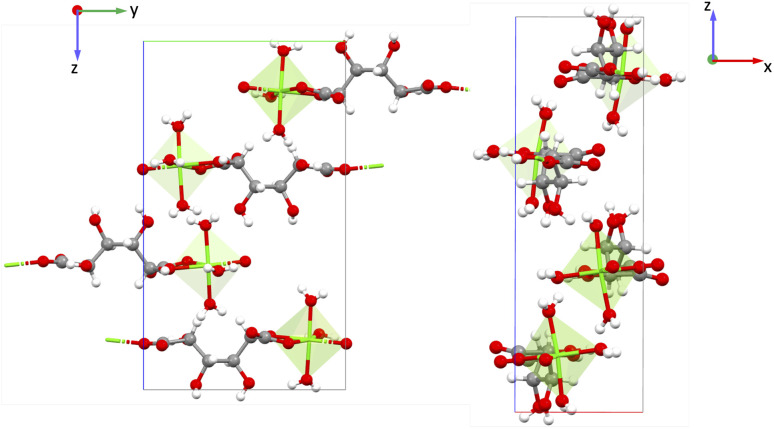
Figure showing AGALUR structure in the *yz* and *xz* planes. Green: magnesium (Mg), gray: carbon (C), red: oxygen (O), white: hydrogen (H).

AGALUR is a metal–organic coordination polymer based on alkaline earth ion Mg^2+^, each coordinated by three water molecules and linked to chains *via* saccharate (Mg(H_2_O)_3_(sacch), Fig. 7), which has a dipole moment.^54^ AGALUR belongs to the non-polar orthorhombic point group ‘222’ where all the non-zero values of *e*_iq_ involve three crystal directions (*x*, *y*, *z*-directions). The piezoelectric norm value ‖*e*_iq_‖_max_ for AGALUR is 0.944 C m^−2^ (the full piezoelectric tensor is shown in the SI in Section 6.3), and the highest value *e* is for *e*_25_ = 0.944 C m^−2^, *i.e.*, the largest polarization is obtained along the *y*-direction when shear is applied along the *xz* plane.

Similar to previously discussed alkali metal ion MOFs (YECPEE and NAHSQU01), the low BEC value (+2.19) of the Mg^2+^ cations does not explain the high ‖*e*_iq_‖_max_ value in AGALUR. We examined the relaxed-ion contribution of the dielectric tensor, which is a measure of the polarizability of the lattice structure and physically represents the movement of ionic charges when subjected to an electric field. In this case, the dielectric tensor shows a strong ionic contribution along the *y*-direction, *ε*_*yy*_ = 8.326 compared to *x*- and *z*-directions (*ε*_*xx*_ = 2.808 and *ε*_*zz*_ = 1.820), indicating atoms along this direction are easily polarizable in the presence of an electric field. The complete clamped-ion and relaxed-ion dielectric tensor is shown in SI Section 7.3. Specifically, saccharate coordination chains are arranged along the *y*-direction (along 2), where the *β*-hydroxyl groups on the chains are easily polarizable. We attribute a large contribution to *e* of AGALUR to the highly polarizable –OH groups on the organic chain, akin to YECPEE and NAHSQU01.

#### 3.4.2 Mo based structures (VAFYOQ and BALFAV01)

**Fig. 8:**
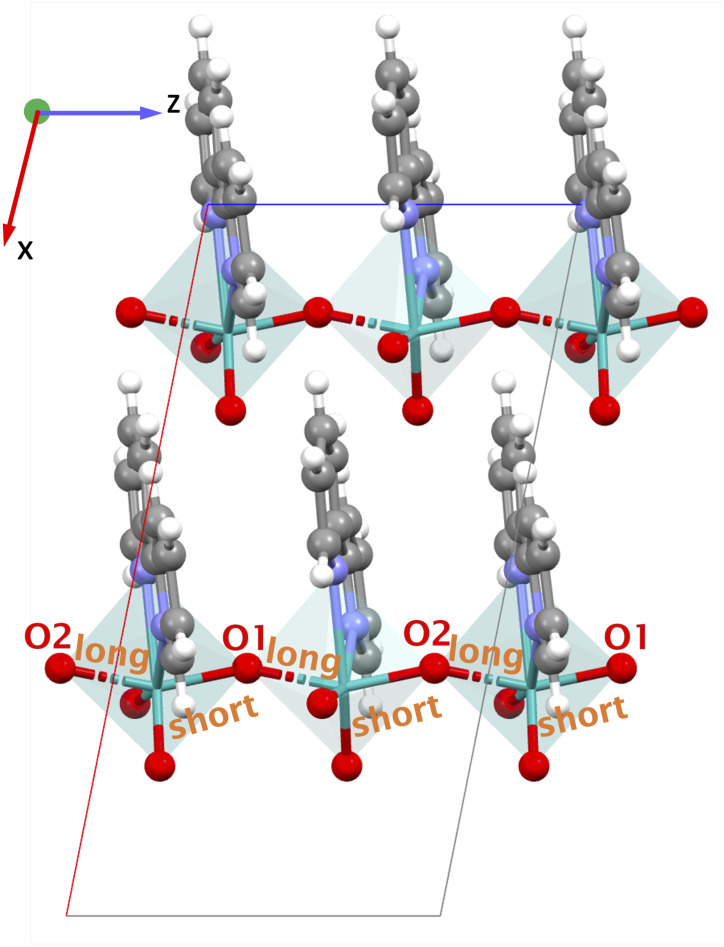
Structures of VAFYOQ showing the *xz* plane and different O1–Mo–O2 short–long–short–long⋯pattern in the bond lengths along the *z*-direction. Teal: molybdenum (Mo), gray: carbon (C), red: oxygen (O), white: hydrogen (H), blue: nitrogen (N).

VAFYOQ is a Mo–MOF belonging to the monoclinic point group ‘*m*’, where all non-zero values of piezoelectric tensor *e*_iq_ involve the *x* and/or *z*-directions. The organic linker in the MOF is polar 2,2′-bipyridine. VAFYOQ (MoO_3_(2,2′-bipy)), Fig. 8) consists of one-dimensional chains of distorted octahedra MoO_4_N_2_ where each Mo is coordinated by two oxo bridging groups shared between the octahedra, two terminal oxo groups, and the two pyridyl nitrogen from the polar 2,2′-bipyridine ligand. The piezoelectric norm value ‖*e*_iq_‖_max_ for this MOF is 1.506 C m^−2^, the highest value in the piezoelectric tensor *e*_33_ is −1.382 C m^−2^ (the full piezoelectric tensor is shown in the SI in Section 6.4) and the average BEC (Z*) of Mo cation in VAFYOQ is +5.43 close to the oxidation state +6. Moreover, VAFYOQ exhibits a high value for 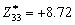 than 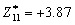 and 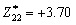. Importantly, along the *z*-direction (direction 3) the Mo-bridging oxo group distances are unsymmetrical (*i.e.*, O1–Mo–O2) giving rise to an alternate short–long–short–long⋯ Mo–O infinite chain with lengths O1–Mo: 1.822 Å and Mo–O2: 2.031 Å.^55^ The dipole moments of the organic ligand are aligned parallel to the *x*-direction. Yet, the piezoelectric tensor component with the highest value is nonethless *e*_33_, thus only involving the *z*-direction. Hence, we attribute this high piezoelectric constant to the high BEC of Mo and polar O1–Mo–O2 chains.

**Fig. 9:**
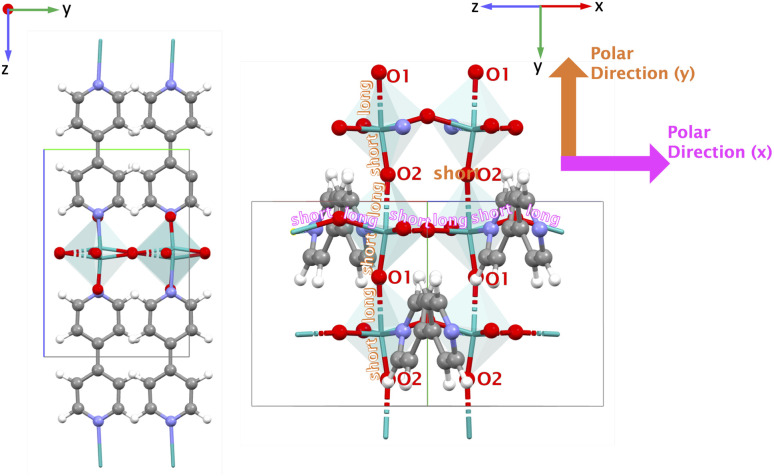
Structures of BALFAV01 (showing the *yz* plane) and different O1–Mo–O2 long–short–long–short pattern in the bond lengths along the *x*- and *y*-directions. Teal: molybdenum (Mo), gray: carbon (C), red: oxygen (O), white: hydrogen (H), blue: nitrogen (N).

BALFAV01 is another Mo–MOF that belongs to the triclinic point group ‘1’ with an apolar organic linker 4,4′-bipyridine. In BALFAV01 ((MoO_3_)_2_(4,4′-bipy), Fig. 9), the Mo inorganic cations are coordinated by one terminal oxo-oxygen, one nitrogen atom from a centrosymmetric 4,4′-bipyridine linker, and four bridging oxygen atoms shared between the Mo octahedra. Along the *z*-direction, the N and terminal oxo-oxygen group are 180° apart, and along the *x* and *y*-directions, a 2-D square grid formed by Mo–O chains is present with two polar directions (*x* and *y*) as shown in Fig. 9. The piezoelectric norm value ‖*e*_iq_‖_max_ for this MOF (polar ‘1’ point group) is 2.763 C m^−2^ and the average Z* for Mo is high around +6.86, close to the oxidation state of +6.0 for Mo. The diagonal values of Z* are 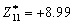, 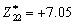 and 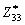 is +4.35. Along the *x* and *y*-directions (direction 1, 2), the O1–Mo–O2 bonds exhibit alternating bond lengths, forming a long–short–long–short⋯pattern.^56^ After geometry optimization of the polar ‘1’ point group structure, the bond lengths are O1–Mo: 1.781 Å (short), Mo–O2: 2.161 Å (long) along the *y*-direction and O1–Mo: 1.821 Å (short), Mo–O2: 2.021 Å (long) along the *x*-direction.

In the experimental structure, the bond length difference between O1–Mo and Mo–O2 is substantially smaller both along the *x*- and *y*-directions. Please note that according to the CSD database the experimental structure BALFAV01 belongs to the monoclinic ‘2’ point group. However, we reduced it to ‘1’, as the simulations indicate that the polar ‘1’ point group structure with this bond-length asymmetry along *x*- and *y*-directions is energetically more favorable than the corresponding ‘2’ point group and non-polar structures. Detailed energy comparisons and structural differences among the polar ‘1’ point group, polar ‘2’ point group and non-polar structures are provided in the SI Section 5.1. Largest values in the piezoelectric tensor components are *e*_11_ = 2.361 C m^−2^ and *e*_22_ = 1.445 C m^−2^, indicating the largest polarization occurs along the polar *x* and *y*-directions when a uniaxial strain is applied along the same *x* and *y*-directions respectively. This is coinciding with the direction of the long–short–long–short⋯Mo–O bond patterns. The full piezoelectric tensor is shown in the SI in Section 6.5).

In both VAFYOQ and BALFAV01, the highest *e* values are seen along the direction of long–short–long–short⋯bonds of O1–Mo–O2. This pattern of long–short–long–short⋯bond lengths, is reminiscent of the structure that gives rise to significant piezoelectricity in typical piezoelectric oxides like BaTiO_3_. In the tetrahedral structure of BaTiO_3_, along the *z*-direction the O1–Ti–O2 bond lengths are unsymmetrical, creating an anisotropic coordination environment around Ti along Ti–O chains.^50^ Additionally, high-born effective charges for Ti (+6.7) are also shown to enhance the piezoelectric constant of BaTiO_3_. Such ceramic piezoelectrics are also ferroelectric, as their polarization is reversible upon application of an external electric field *via* alteration of the O—(short)—Ti—(long)—O pattern. The similar O—(short)—M—(long)—O pattern responsible for a high piezoelectric response in the discussed Mo–MOFs could mean they may be potential ferroelectric materials. This would make the materials particularly interesting for piezoelectric applications, as such materials are more easily processable. Specifically, it means that a polycrystalline device can be poled to have an overall polarization *via* the ferroelectric effect.

##### 3.4.2.1 SHG of Mo–MOF VAFYOQ

**Fig. 10:**
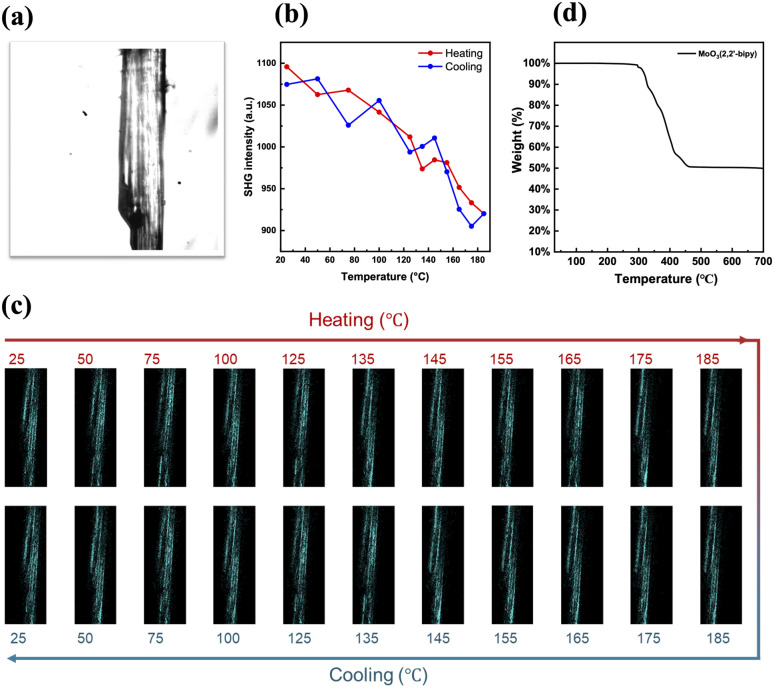
(a) Optical image of VAFYOQ crystal under observation; (b) SHG intensity change upon heating and cooling when incident and detected light polarization is along O–Mo–O chains – the long direction of the crystals – and the (c) corresponding SHG images, and (d) TGA analysis of VAFYOQ under air.

To substantiate this possibility, we investigated experimentally the occurrence of a Curie temperature *via* SHG-microscopy. A Curie temperature is a defining feature of a ferroelectric material at which it undergoes a transition from the ferroelectric polar phase to a paraelectric phase where it loses its spontaneous polarization. At the Curie temperature, the material would thus lose its SHG-activity. Of the Mo–MOFs we managed to synthesize VAFYOQ (MoO_3_(2,2′-bipy)), which yielded optically transparent crystals that show consistent SHG-activity along the whole crystal (Fig. 10a). Do note that in this Mo–MOF structure, the 2,2′-bipy are arranged in a polar manner, which can't be reversed without bondbreaking and making. Hence, we expect to only see a decrease in SHG with temperature but no disappearance thereof. In Fig. 10b and c, it can be seen that indeed the SHG-intensity is decreasing between 100 °C to 185 °C, when both incident and detected light are linearly polarized parallel to the O–Mo–O bond direction. This is before the thermal composition, as indicated by no weight loss thermogravimetric analysis (TGA) (see Fig. 10d before 300 °C), and the reversibility: the original SHG-intensity is recovered upon cooling (see Fig. 10d). Interestingly, when the incident and detected light is polarized perpendicularly to the O–Mo–O bond direction, the SHG-intensity remains constant until 185 °C (see Fig. S9 in SI).

It is thus likely that the lowering of the SHG for light polarized aligned with the O–Mo–O direction is due to a decrease in the difference between the bond length distances of the different Mo–O bonds. While this is not a conclusive proof of ferroelectricity, this does show the malleability of these bond lengths *via* external stimuli. The observation is in line with the behavior of inorganic MoO_3_, for which it was recently shown that it can have polar and ferroelectric phases based on different bond lengths within the O–Mo–O motif.^57,58^

#### 3.4.3 Sb based structure (EHOHOY)

**Fig. 11:**
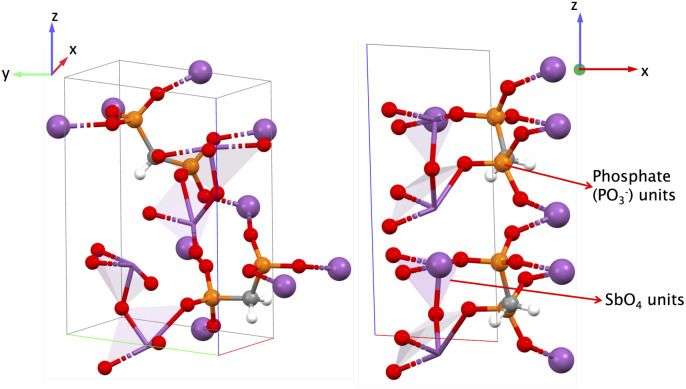
Figure (left): EHOHOY structure with trigonal bipyramidal SbO_4_ and PO_3_(CH_2_) units and (right): structure indicating SbO_4_ and PO^−^_3_ units along the *x*-direction. Plum: antimony (Sb), gray: carbon (C), red: oxygen (O), white: hydrogen (H), orange: phosphorous (P).

This MOF (Sb_2_O(O_3_PCH_2_PO_3_) belongs to point group ‘*m*’ where all non-zero values of *e*_iq_ involve the *x* and/or *z*-directions. The structure of EHOHOY, shown in Fig. 11, consists of Sb^3+^ cation coordinated to methylenediphosphonate (O_3_PCH_2_PO_3_)^4−^, a ligand with a dipole moment. The structure exhibits a three-dimensional network comprising of trigonal bipyramidal SbO_4_ units and tetrahedral [PO_3_(CH_2_)] units.^59^ Polar bipyramidal units of SbO_4_ and polar phosphonate units (PO^−^_3_) are partially aligned along the *x*-direction.

The piezoelectric norm ‖*e*_iq_‖_max_ is 1.159 C m^−2^ and the highest *e* value is *e*_11_ = −1.076 C m^−2^, indicating the greatest polarization along the *x*-direction when a strain is also applied along the *x*-direction (the full piezoelectric tensor is shown in the SI in Section 6.6). In this MOF, there are two distinct cations, Sb and P, each with BEC Z* of +3.93 and +3.60, respectively and BEC Z* of O and C are −2.04 and −0.94 respectively. There are multiple Sb–O–P–O–Sb…bonding pathways in the structure aligned partly along the *x*-direction and in the *xz*-plane. Similarly, there are multiple Sb–O–P–C–P–O–Sb…bonding pathways mainly along the *y*-direction and *yz*-plane. The bond lengths of Sb–O vary between 1.963 Å and 2.377 Å, and P–O vary from 1.532 Å to 1.560 Å. We attribute the high *e*_11_ value for this MOF, to the Sb–O–P–O–Sb–O–P–O…bonding pathway of varying bond lengths and distinct BECs of both Sb and P leading to a net polarization. For pathway Sb–O–P–C–P–O–Sb…, we observed varying bond lengths for Sb–O and P–O as mentioned before, but the same bond length for C–P = 1.806 Å. Given its orientation in the structure along the *yz*-plane, we hypothesize that this latter bonding pathway is not a major contributor to the highest *e* value for this structure, *i.e.*, *e*_11_.

#### 3.4.4 Zn based structures (GAXQAZ and UVAROZ)

**Fig. 12:**
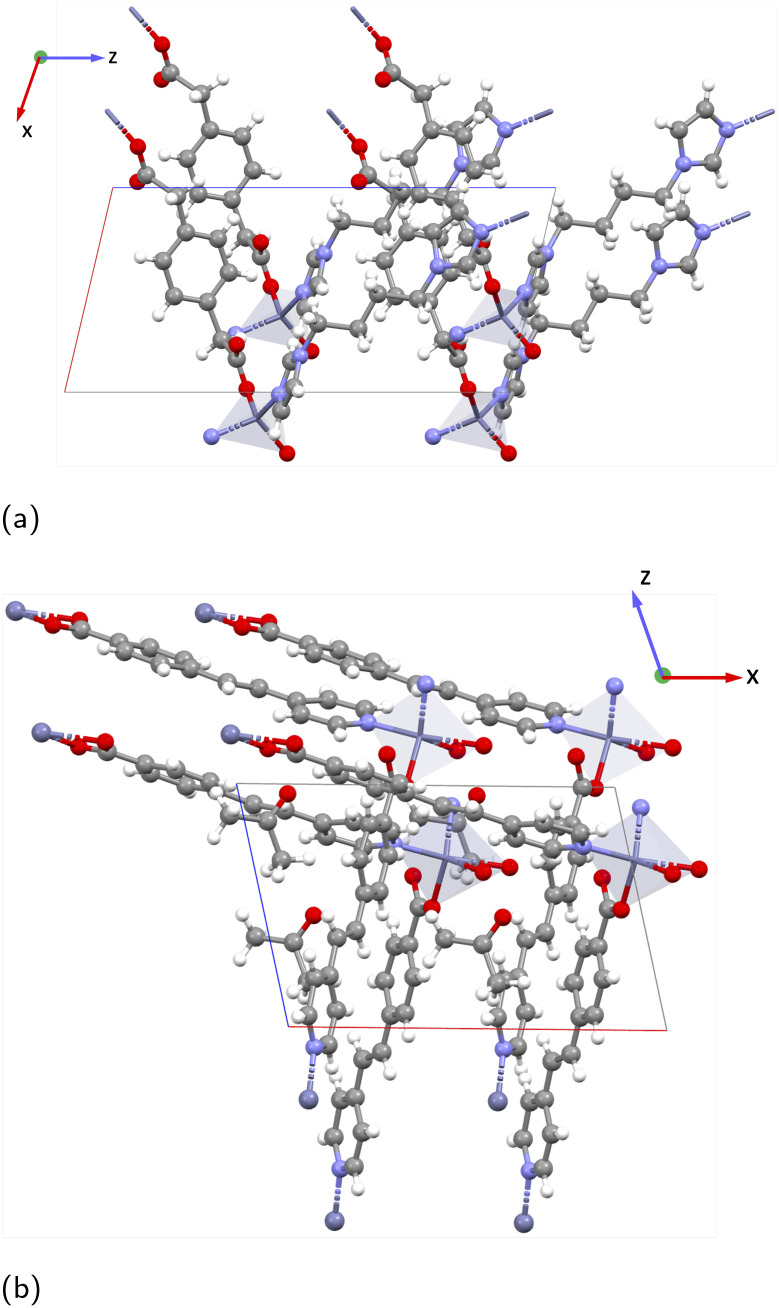
Structures of (a) GAXQAZ (showing the *xz* plane) and (b) UVAROZ (showing the *xz* plane). Tetrahedral: zinc (Zn), gray: carbon (C), red: oxygen (O), white: hydrogen (H), blue: nitrogen (N).

Both these MOFs are Zn-based, crystallizing in the monoclinic ‘*m*’ point group, thus all the non-zero values of the piezoelectric tensor *e*_iq_ involve the *x* and/or *z*-directions. GAXQAZ (Zn(*p*-pda)(biim-4)) has a five-fold interpenetrated diamondoid structure in three dimensions with 1,4-phenylenediacetate acid (*p*-pda) and 1,1’-(1,4- butanedidyl)bis-(imidazole) (biim-4) linkers, shown in Fig. 12a). UVAROZ (Zn(pyeb)_2_) has a 7-fold interpenetrated three-dimensional diamondoid structure with 4-[2-(4-pyridyl)ethenyl] benzoic acid (pyeb) linker, shown in Fig. 12b).^60,61^ The piezoelectric norm value for GAXQAZ is 1.092 C m^−2^ and for UVAROZ it is 1.066 C m^−2^. In both cases, Zn is coordinated with the two O and two N atoms from the organic linkers, creating a polar coordination environment around Zn^2+^.

The BECs of Zn^2+^ in GAXQAZ and UVAROZ are +2.13 and +2.71, respectively, hence not very high in magnitude. GAXQAZ has five high *e*_ip_ values that are along 11, 31, 13, 15 and 35 with magnitudes between ± 0.4 and ±0.5 C m^−2^. This indicates that the largest polarization is along the *x*- and *z*-directions when a strain is applied along the *x*-, *z*-directions, and *xz*-plane. For UVAROZ, *e*_13_ = −0.724 C m^−2^ is the *e*_ip_ with the highest magnitude, hence the largest polarization is along the *x*-direction when a strain is applied along the *z*-direction. The full piezoelectric tensor of these MOFs is shown in the SI in Section 6.7 and 6.8. Fig. 12 shows the *xz* (13) plane of the GAXQAZ and UVAROZ. The high piezoelectric response in GAXQAZ and UVAROZ does not appear to arise primarily from the Born effective charges (BEC) of the inorganic cations. Instead, a likely contributing factor could be the polar coordination environment of the Zn^2+^ cation, along with the alignment of polar ligands in a net polar manner.

### 3.5 Comparison of piezoelectric and mechanical properties of MOFs with existing piezoelectrics

Here, we discuss and compare the piezoelectric properties (*e*, *d*), mechanical compliance (*s*) and dielectric constants of a few MOFs in this work with some existing inorganic, organic and hybrid perovskite piezoelectrics. Among the eight MOFs previously discussed with ‖*e*_iq_‖_max_ ≥ 1.0 C m^−2^, we calculated the piezoelectric tensor *d* for four MOFs; two Mo–MOFs (BALFAV01 and VAFYOQ), one of the alkali metal based MOF (NAHSQU01) and Sb-based MOF (EHOHOY). We exclude here the Zn MOFs, as their interpenetrated structure adds additional uncertainty to the computational values of mechanical compliance (*s*). The methodology for computing *d* for these MOFs and the complete piezoelectric tensor *d*_ip_ is described in SI Section 2.5 and 8 respectively.

We compare the piezoelectric tensor values (*e*_ik_, *d*_ik_) and mechanical compliance (*s*_pq_) of various materials in Table 1, which includes inorganics (PZT, LiNbO_3_), organic piezoelectrics (PVDF-TrFE), hybrid inorganic-organic perovskites (TMCM– MnCl_3_, TMCM–CdCl_3_), metal-free perovskites (MDABCO–NH_4_–X_3_), and MOFs in this work. In Table 1, we report the range of *e*_ik_, *d*_ik_, and *s*_pq_ tensor components (minimum to maximum values) for each material, rather than a single value in the tensor. In terms of the piezoelectric constant *e*_ik_, inorganics especially PZT exhibits the highest value on the order of 10^1^ C m^−2^, followed by MOFs in this work (10^0^ C m^−2^), while organic polymer PVDF-TrFE and hybrid perovskites display significantly lower responses (10^−1^ C m^−2^). Within the MOFs discussed here, *e*_ik_ for Mo–MOFs and Sb–MOF (∼1 C m^−2^ to 2 C m^−2^) is larger than PVDF-TrFE (0.19 C m^−2^), hybrid perovskites (0.35 C m^−2^), and is in the same range of values as LiNbO_3_.

**Table 1:** Comparison of piezoelectric tensors *e*_ik_, *d*_ik_, compliance tensor *s*_pq_ and dielectric constant *ε*_r_ of some inorganic, organic, hybrid piezoelectrics with MOFs in this work^a^

Material type	Material	|*e*_ik_| (C m^−2^)	|*s*_pq_| (TPa^−1^)	|*d*_ik_| (pC N^−1^)	*ε*_r_
Inorganic	PZT	1.96–10.9	1.13–17.54	122, 285, 495	1300
Inorganic	LiNbO_3_	0.08–2.90	2.0–24.0	6, 69	34
Organic	PVDF-TrFE	0.07–0.19	90.90–714.28	7–50	12
Inorganic–Organic perovskites	TMCM– MnCl_3_			185	
Inorganic–Organic perovskites	TMCM– CdCl_3_			220–240	35
Metal–Free perovskites	MDABCO–NH_4_–X_3_ [X = Cl, Br, I]	0.05–0.35	12.5–650	14.4–240	<40
MOFs in this work	NAHSQU01	0.03–0.61	5.24–102.72	0.76–11.05	
MOFs in this work	VAFYOQ	0.06–1.42	4.79–159.87	3.40–50.37	
MOFs in this work	BALFAV01	0.01–2.13	0.05–110.89	0.11–86.25	7.6
MOFs in this work	EHOHOY	0.01–1.12	3.81–93.01	0.36–42.09	

a Ref. 9,48, and 62 for PZT; 9,26,27, and 43 for LiNbO_3_; 10 for PVDF-TrFE; 11,13, and 14 for inorganic–organic perovskites; 12 for metal-free perovskites.

Organic piezoelectric PVDF-TrFE and metal-free perovskites stand out with exceptionally high mechanical compliance values *s*_pq_, indicating their soft and flexible nature. In contrast, the inorganics PZT and LiNbO_3_ are significantly stiffer. The MOFs fall in the intermediate range, with compliance values that bridge the gap between stiff inorganics and highly flexible organics. Finally, the values of the piezoelectric tensor *d*_ik_ are the highest for PZT followed by inorganic-organic perovskites and metal-free perovskites with values of the same order of magnitude (10^2^ pC N^−1^) as PZT. For LiNbO_3_, PVDF-TrFE, and MOFs in this work, the values are of the order 10^1^ pC N^−1^ and one order of magnitude lower than PZT. Within the MOFs studied in this work, the Mo–MOF BALFAV01 has the highest *d*_ik_ (∼86 pC N^−1^), which is greater than the piezoelectric response of inorganic LiNbO_3_ (69 pC N^−1^) and organic PVDF–TrFE (50 pC N^−1^).

In addition to the piezoelectric constants *e* and *d* and the mechanical compliance *s*, a lower dielectric constant is advantageous to maximize efficiency, as indicated in eqn (2). Guest-free MOFs usually have very low dielectric constants (2.3 to 6.0)^28–36^ due to their inherent high permanent porosity in the structure, which is lower than even organic piezoelectric materials. Due to the relatively high inorganic fraction and low porosity of MOFs of the Mo-structures discussed in this work, we additionally computed the value of the dielectric constant for the Mo–MOF (BALFAV01) to be around 7.6. Even this value is still lower than that of organic piezoelectric PVDF-TrFE, and one or more orders of magnitude lower compared to the other material classes (Table 1). The estimated FoM of BALFAV01 calculated using the highest *d*_ik_ value (86.25 pC N^−1^) and computed dielectric constant (7.6) in this work is around ∼978/*ε*_0_ (pC N^−1^)^2^. For PZT, the estimated FoM from the values of *d* and the dielectric constant in Table 1 is around ∼188/*ε*_0_ (pC N^−1^)^2^. Overall trends of piezoelectric, mechanical, and dielectric properties suggest that, while MOFs may not match traditional inorganic materials in piezoelectric properties, their high compliance combined with very low dielectric constants could be advantageous for flexible energy harvesting applications.

## 4 Conclusions

In this work, we investigated the piezoelectric properties of the experimentally synthesized 1263 MOF structures through high-throughput periodic DFPT calculations. The total piezoelectric tensor (*e*_iq_) and the two contributions of *e*_iq_: clamped-ion (*e*^0^_iq_) and internal strain tensors (*e*^int^_iq_) along with the born effective charges (BEC) for all the atoms in the structure are obtained from the piezoelectric calculations. Our results show that a high value for *e* is more likely in polar MOFs than non-polar MOFs. For the MOFs in this study, the internal strain contribution is strongly correlated with the total piezoelectric constant *e* and is the dominant factor in obtaining a high *e* value. Unlike traditional piezoelectrics of ABO_3_ type, where a high born effective charge (BEC) of metal cations leads to a high *e*, the driving factor for obtaining a high *e* in MOFs is much more complex and structure dependent. It includes the combined effect of BEC of all atoms (including inorganic cations of the secondary building units and atoms in the organic linkers of the MOF) and the relative changes in their atomic positions.

Hence, for MOFs that show high values of piezoelectric constant *e* ≥ 1.0 C m^−2^, we highlighted the key structural factors contributing to their high performance. Based on those observations, we summarize a series of guidelines for MOF structures (not exhaustive) that can lead to a high *e* value.

1. The structure belongs to a polar point group.

2. Mo–MOFs in this work (VAFYOQ and BALFAV01) with O1–Mo–O2—⋯bonds have a long–short–long–short⋯bond length patterns along a specific direction organized in a polar manner. Additionally, Mo cations also have a high born effective charge (BEC) of ∼ + 6. Hence, having a high born effective charge (BEC) for the inorganic cations together with an anisotropic coordination environment around them due to the long–short–long–short⋯pattern of bonds that are organized in a polar manner can lead to a high *e* value.

3. Similarly, the coexistence of different inorganic cations within the same structure with varying Born effective charges (BEC) like Sb and P in EHOHOY, can lead to patterns like Sb–O–P–O–Sb–O–P–O…with varying bond length leading to a high net polarization.

4. The presence of polar molecules such as water, directly bonded to the inorganic cation and aligned along a specific direction (YECPEE, NAHSQU01 and AGALUR).

5. A very polar coordination environment around the inorganic cation together with polar organic ligands aligned in a polar manner (GAXQAZ and UVAROZ). For example, if the inorganic cation is coordinated to two N atoms and two O atoms in a tetrahedral coordination, a polar coordination environment is possible.

The Mo–MOFs (class 2 here), could potentially be ferroelectric as well, as the mechanism of their piezoelectric behavior, namely unequal O–Metal atom–O bond lengths, is the same as that of quintessential inorganic ferroelectrics like BaTiO_3_, where the polarity can be reversed *via* an electric field causing the alternation of the O—(short)—M—(long)—O bond patterns. We indeed found experimental indication, *via* decrease in the SHG-activity of VAFYOQ with temperature specifically for light polarized along the O–Mo–O bond direction, that the O—(short)—Mo—(long)—O bond lengths are malleable, and may have potential for ferroelectric behavior. The latter is very relevant for the processability of materials for piezoelectric applications, as the ferroelectric property allows the poling of polycrystalline films into an overall polarization.

Finally, we compared the piezoelectric properties *e* and *d*, and the mechanical compliance *s* of selected MOFs in this work with existing inorganic, organic, and hybrid piezoelectrics. MOFs exhibit intermediate piezoelectric and mechanical properties compared to those of inorganic materials, organic polymers, and hybrid perovskites. However, owing to their inherent porosity in the structure, MOFs have a very low dielectric constant compared to existing piezoelectrics, making them efficient for piezoelectric energy harvesting. By establishing structure–property relationships for piezoelectric properties in metal–organic frameworks (MOFs), this work provides a valuable guide for the rational design of novel and previously unexplored piezoelectric MOFs, thereby accelerating the discovery of efficient materials for piezoelectric energy harvesting applications.

## Author contributions

S. M. conceived and designed the project, performed the computational work and analysis, and drafted the manuscript. C. H. carried out the experimental work and contributed the experimental part of the manuscript. M. V. and F. G. provided scientific guidance and contributed to interpretation of the results. M. V. conceived the study and supervised the research. All authors edited, reviewed and approved the final version of the manuscript.

## Conflicts of interest

There are no conflicts to declare.

## Supplementary Material

TA-014-D5TA09332E-s001

TA-014-D5TA09332E-s002

TA-014-D5TA09332E-s003

TA-014-D5TA09332E-s004

TA-014-D5TA09332E-s005

TA-014-D5TA09332E-s006

TA-014-D5TA09332E-s007

## Data Availability

The data supporting this article have been included as part of the supplementary information (SI). Supplementary information: (1) Detailed theory of piezoelectricity, methods used for the computation of piezoelectric tensors *e* and *d*, the full piezoelectric tensors, and the Born effective charges of top performing MOFs in this work [SI.pdf]. (2) Structural details, piezoelectric tensor *e*_iq_, piezoelectric norm ‖*e*_*iq*_‖_max_, average BEC of inorganic cation for MOFs with converged and reliable results [Totale_BEC_Structuredetails.xlsx]. (3) Highest *e*_iq_ values of the top 8 MOFs [Top8MOFs_highestevalues.xlsx]. (4) Clamped and internal strain piezoelectric tensors for all the MOFs computed in this work [Clamped_eiq_allMOFs.xlsx, InternalStrain_eiq_allMOFs.xlsx]. (5) Convergence tests for ENCUT and KPOINTS values for high throughput piezoelectric tensor calculations [Encut_and_*k*points_convergence.xlsx]. (7) Piezoelectric tensor, elastic tensor and compliance tensor of top ‘*e*’ MOFs [TopeMOFs_piezoelectricconstant_d_CRYSTAL17.xlsx]. See DOI: https://doi.org/10.1039/d5ta09332e. The raw data of the DFT calculations and python scripts used for calculation automation and plotting are made available on 4TU data repository at https://doi.org/10.4121/eb4e3397-140d-4e8f-aceb-cd2a7520f309.
