# Supplementary material for: Computational screening of piezoelectric constants in metal–organic frameworks: design principles and ferroelectric-like bond modulation
Source: J Mater Chem A Mater. 2026 Feb 24;14(23):14311–23. doi: 10.1039/d5ta09332e (PMC12951834; doi:10.1039/d5ta09332e)
Supplement: TA-014-D5TA09332E-s004 [file TA-014-D5TA09332E-s004.pdf]

Supplementary Information (SI) for Journal of Materials Chemistry A.
This journal is © The Royal Society of Chemistry 2026

Supporting Information

Computational Screening of Piezoelectric Constants in Metal–Organic Frameworks: Design Principles and Ferroelectric-Like Bond Modulation

Srinidhi Mula, Chunyu Huang, Ferdinand Grozema, and Monique A. van der Veen*

Department of Chemical Engineering, Technische Universiteit Delft, Delft, 2629HZ, The Netherlands

E-mail: m.a.vanderveen@tudelft.nl

1 Theory

Piezoelectricity is mathematically described in the IEEE standard for piezoelectricity[1] by a set of four constitutive equations that describe the response of a piezoelectric material to a mechanical load (stress/strain) and electric fields. Of relevance in this work are the sets of constitutive equations shown in equation 1.

$$
\begin{aligned}
D_i &= e_{ikl}S_{kl} + \varepsilon^S_{ij}E_k \\
S_{ij} &= s^E_{ijkl}T_{kl} + d_{kij}E_k
\end{aligned}
\tag{1}
$$

where D, S, ε, E, s, T represent electric displacement [C/m^2] strain tensor (dimensionless), dielectric constant [F/m], electric field [V/m], elastic compliance tensor [m^2/N] and stress tensor [N/m^2] respectively. e and d are piezoelectric tensors with units [C/m^2] and [pC/N] respectively. Note that of importance in piezoelectric applications is the value of d which

effectively links the applied electrical field to the deformation of the material (strain S), relevant for transducers, or the applied stress (T) to the electric displacement (D) which can be stored as energy in piezoelectric energy harvesting. This relation between electrical and mechanical properties is pictorially shown by Heckmann diagram in Figure S1 adapted from the book by JF Nye.[2]

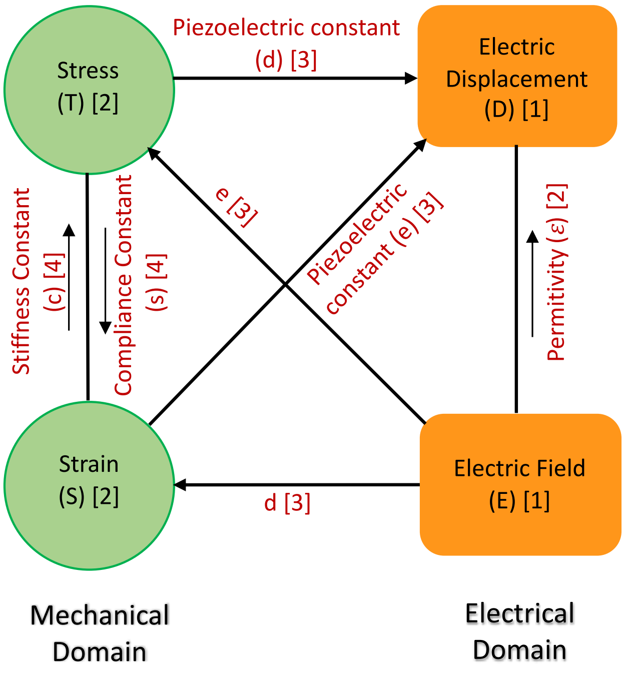

Figure S1: Part of Heckmann diagram showing the relation between mechanical and electrical properties, various variables, and their symbols. The tensor rank of the properties and variables is shown in square brackets.

We will use Voigt notation for the fourth-order elastic (s^{E}_{ijkl}) and third-order piezoelectric tensors (e_{ikl}, d_{kij}) where the indices are given in compressed matrix notation instead of tensor notation. This means that the indices ij or kl where $i, j, k, l = 1, 2, 3$ are replaced by p or q and pairs of contracted cartesian directions $p, q = 1, 2, 3, 4, 5, 6$ where 11:1; 22:2; 33:3; 23, 32:4; 13, 31:5; 12, 21:6. The piezoelectric tensor d_{ip} can be computed from the piezoelectric constant e and the elastic compliance constant s using $d_{ip} = e_{iq}s^{\mathrm{E}}_{qp}$. Computationally, the piezoelectric constant e_{iq} is calculated as the first derivative of the magnitude of the polarization P induced by strain at constant electric field E, $e_{iq} = \left(\frac{\partial P_i}{\partial \varepsilon_q}\right)_E$. In this work, we compute the

piezoelectric tensor, e_{iq} which relates the polarization generated in the material on application of a deformation or strain to the material.

To understand the microscopic origin of piezoelectricity in a material, the piezoelectric tensor e_{iq} can be further separated into two parts: the clamped-ion (e_{iq}^{0}) and the internal strain (e_{iq}^{int}) contributions.[3,4] For a general case, the piezoelectric constant e_{iq} is shown in equation 2.[5]

$$\begin{aligned} e_{iq} &= \frac{\partial P_i}{\partial S_q} \\ e_{iq} &= \left(\frac{\partial P_i}{\partial S_q}\right)_x + \sum_s \sum_p \left(\frac{\partial P_i}{\partial x_{sp}}\right)_{S_q=0} \left(\frac{\partial x_{sp}}{\partial S_q}\right)_{S_q=0} \\ e_{iq} &= e_{iq}^{0} + e_{iq}^{\mathrm{int}} \end{aligned} \tag{2}$$

The clamped ion contribution (e_{iq}^{0}) is the change in polarization P_i due to the reorganization of electron density with external strain S_q, while the fractional coordinates x are kept constant in the strained unit cell. The internal strain contribution (e_{iq}^{int}) provides the change in polarization P_i where the atoms are allowed to relax in the strained unit cell in response to the strain S_q. e_{iq}^{int} is related to Born effective charges (BEC, $Z_{s,ip}^{*}$) as shown in equation 3.[5] The BEC is a dynamical charge introduced by Max Born and Maria Goppert Mayer and it is a second rank tensor. It refers to a change in polarization induced by an atomic displacement under the condition of zero macroscopic electric fields.[6] Thus, the internal strain contribution is obtained by summing the product of BECs, a second-order tensor ($Z_{s,ip}^{*}$), and the respective relaxation coefficient ($\frac{\partial x_{sp}}{\partial S_q}$) over the total number of atoms s and direction of atom displacement p.

$$e_{iq}^{\mathrm{int}} = \frac{ea}{V} \sum_s \sum_p Z_{ip}^{*} \frac{\partial x_{sp}}{\partial S_q} \tag{3}$$

The general form of piezoelectric tensor e_{iq} is $\begin{bmatrix} e_{11} & e_{12} & e_{13} & e_{14} & e_{15} & e_{16} \\ e_{21} & e_{22} & e_{23} & e_{24} & e_{25} & e_{26} \\ e_{31} & e_{32} & e_{33} & e_{34} & e_{35} & e_{36} \end{bmatrix}$ and born effective charge for an atom Z_{ij}^{*} is $\begin{bmatrix} Z_{11}^{*} & Z_{12}^{*} & Z_{13}^{*} \\ Z_{21}^{*} & Z_{22}^{*} & Z_{23}^{*} \\ Z_{31}^{*} & Z_{32}^{*} & Z_{33}^{*} \end{bmatrix}$.

2 Computational Methods

2.1 Database Selection

We start from the QMOF database (v13), a computationally ready database consisting of 20425 MOF structures.[7] Various criteria as listed below were considered for the candidates selection in this work:

1. Only experimentally deposited structures from the Cambridge Structural Database (CSD)[8] are considered.

2. For a material to be piezoelectric, it must be non-centrosymmetric. Hence, noncentrosymmetric structures, either polar or nonpolar were chosen.

3. MOFs with metals like Ag, Au, and most of the lanthanide metals are excluded. MOFs with exotic metals Ag and Au were excluded because of the low stability of MOFs with Ag and Au. Among the lanthanides, MOFs with only La, Ce, and Yb are included in the database.

4. Originally in the QMOF database, all the experimental structures from CSD database were reduced to "1" point group (no symmetry), and the optimization was performed. However, in this work, since the piezoelectric tensor is dependent on the point group of the structure, the structures were re-optimized while considering the symmetry. These are referred to here as symmetrized QMOF structures.

   MOFs which have the same space group in both the original CSD and symmetrized QMOF structures were chosen. This is to ensure that the space group of symmetrized QMOF structures after making them computational ready is the same as that of the CSD experimental structure.

5. Lastly, the number of atoms per primitive cell in the MOF structure is limited to $<=$ 150 atoms.

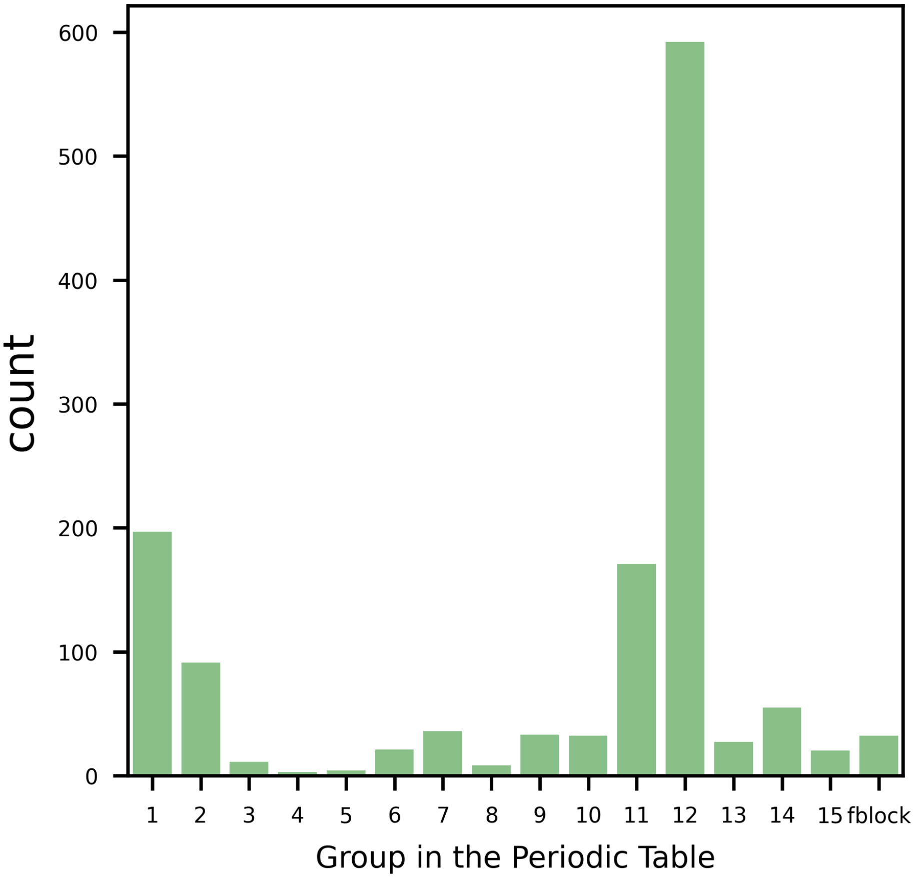

Figure S2: Distribution of inorganic cations in the secondary building unit for the selected MOFs based on the group they belong to in the periodic table.

Considering all the above criteria, around 1608 MOF structures are obtained as starting candidates for the piezoelectric calculations. Among these, 442 MOFs belong to non-polar point groups and 1166 MOFs belong to polar point groups. Figure S2 shows a distribution of the inorganic cation in the secondary building unit of the selected MOF candidates for the high-throughput calculations based on the group in the periodic table they belong to. The database is dominated by MOFs with metal ions from Group 12 (i.e., Zn, Cd) followed by Group 1 (i.e., Li, Na, K) and Group 11 (i.e., Cu).

2.2 Computational Settings for the Computation of Piezoelectric Tensor e_{iq}

Piezoelectric DFT calculations were carried out in two steps. As mentioned earlier in section 2.1, in the QMOF database all structures were reduced to "1" point group (no symmetry). However, since the piezoelectric tensor is constrained by the point group symmetry of the crystal, the structural symmetry is explicitly taken into account in the calculations presented in this work. In the first step, we re-optimize the structures from the QMOF database while considering the symmetry of the unit cell and referred to as symmetrized QMOF structures. In the second step, the piezoelectric constant of the symmetrized QMOF structures is calculated using the Density Functional Perturbation theory (DFPT).[9–11] DFPT allows computing useful physical properties like elastic and piezoelectric constants, Born effective charges, etc., as a second-order linear response to different perturbations like atomic displacements, electric fields, etc.

All the plane-wave periodic DFT calculations in this work were done using Vienna Ab Initio Simulation Package (VASP v5.4.4).[12,13] We used Perdew, Becke, and Ernzerhof (PBE) GGA exchange-correlation functional[14] for all the calculations. Grimme's D3 dispersion corrections and Becke-Johnson damping[15,16] was included for van der Waals dispersion corrections. VASP-recommended projector augmented wave (PAW) pseudopotentials were considered for all elements. (Except for Yb, Yb_3 pseudopotential was used rather than Yb_2 since Yb(III) is more common).[13,17] For the plane wave cutoff energy and the number of k-points, we performed a convergence study of the piezoelectric tensor with these parameters for two MOFs and this is included in section 2.3. Based on the convergence study, we use 520 eV as plane wave energy cutoff and a k-point density of $\sim$ 1000 per number of atoms for all calculations in this work.

2.3 Benchmarking of VASP Parameters with Piezoelectric Tensor

Convergence tests were carried out for two MOFs with CCDC codes REDMUL and VESBEC01. They are Y-based (Yttrium) and La-based (Lanthanum) MOFs respectively. Furthermore, convergence tests were also performed for two ZIFs (ZIF-8 and CdIF-1) that were previously studied computationally for their piezoelectric properties.[18]

2.3.1 Convergence of piezoelectric tensor with ENCUT values

For the convergence tests in both the optimization and the piezoelectric calculation steps, we use two ENCUT values of 520 eV and 700 eV while keeping the density of k-points to 1000 per number of atoms. In the optimization step, the energy difference per atom between ENCUT values of 500 eV and 700 eV is below 0.003 eV, demonstrating acceptable energy convergence with respect to the plane-wave cutoff. We then examine the effect of the ENCUT values on the norm of the piezoelectric tensor. For both MOFs, with the change in encut the piezoelectric norm values ($\|e_{iq}\|_{max}$) remain almost the same. Hence, we consider ENCUT=520 eV for all high-throughput calculations.

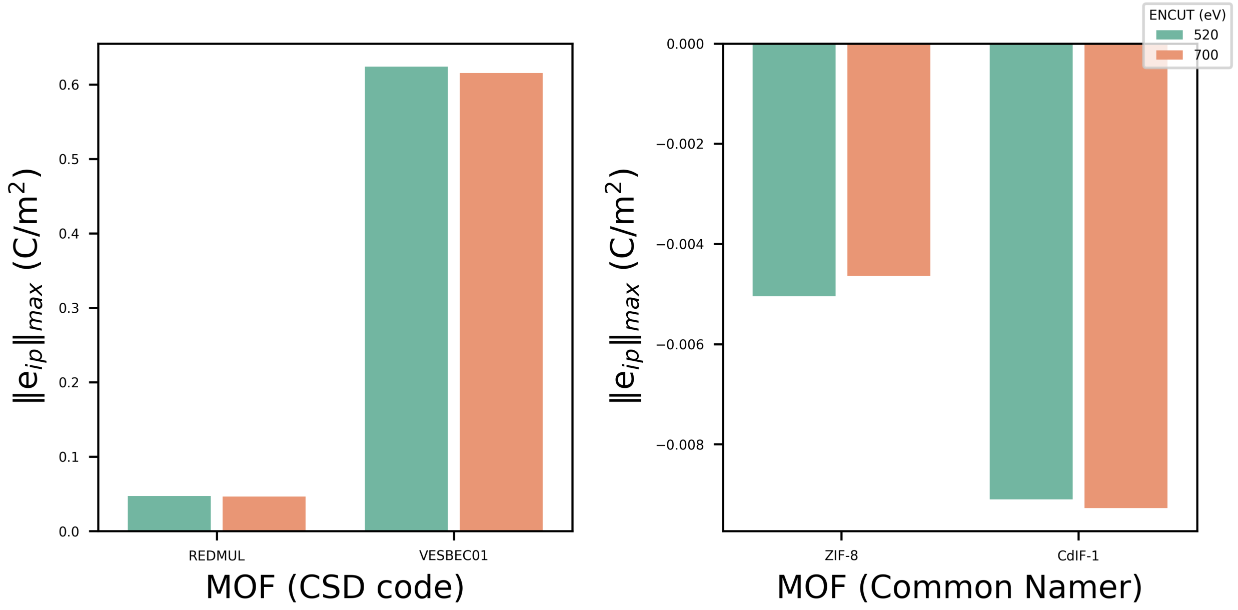

Figure S3: Piezoelectric constant $\|e_{iq}\|_{max}$ values at two different ENCUT values of 520 eV and 700 eV for MOFs in the QMOF database (left) and for ZIFs (right).

2.3.2 Convergence of piezoelectric tensor with k-points values

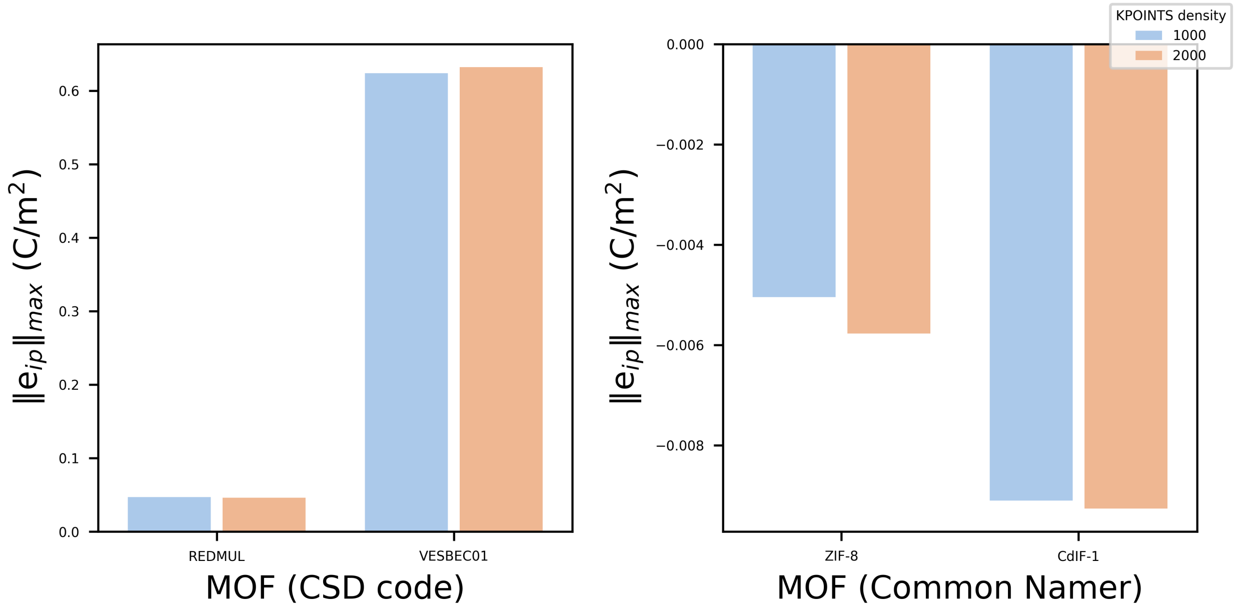

Figure S4: Piezoelectric constant $\|e_{iq}\|_{max}$ values at two different KPOINTS of 1000 and 2000 for MOFs in the QMOF database (left) and for ZIFs (right).

In this convergence test, the encut value 520 eV is kept constant and we vary the k-points density to 1000 and 2000 per number of atoms. In the optimization step, the energy difference per atom between k-point values of 1000 and 2000 is below 0.001 eV, demonstrating energy convergence with respect to the k-points. The change in the values of the 'e' norm with the change in k-point density is less than 1.3%, therefore, we use the k-point density of 1000 per number of atoms in the next calculations.

2.4 Consistency Checks for the DFT Calculations

With the finalized ENCUT, k-points values, and other parameters for the calculations, we geometry-optimize the selected MOF structures followed by a DFPT calculation, resulting in the piezoelectric tensor. Several consistency checks at different stages in the calculations are used to detect possible errors, non-converged results, and obtain only reliable piezoelectric tensors. The filters chosen are as follows:

1. Checks on the net magnetic moment:
   (a) The final magnetic moment obtained in the calculations (where symmetry is taken into consideration) after geometry optimization should be the same as the net magnetic moment which is deposited in the QMOF database for MOF structures (where symmetry is not considered).
   (b) The net magnetic moment at the end of the geometry optimization step and the piezoelectric calculation step should be equal. We expect it to be the same theoretically because the atoms are displaced by a very small strain in the piezoelectric calculations within the harmonic limit, which shouldn't change the net magnetic moment of the entire structure.
2. Checks on the calculated piezoelectric tensor: The following filters are based on symmetry considerations concerning the point group of the structure.
   (a) By virtue of symmetry, $|e_{iq}|$ should be zero for some components in the piezoelectric tensor. This is checked separately in clamped ion and internal strain tensor to be, $\leq 0.1\%$ i.e, $\left|e^{0}_{iq}\right| \leq 0.001\,\mathrm{C/m^2}$ and $\left|e^{int}_{iq}\right| \leq 0.001\,\mathrm{C/m^2}$.
   (b) For point group symmetries that have two components e_{ip} and e_{iq} to be equal, these are checked to be within 0.1% in both the clamped ion and internal strain tensors, i.e., $\left|e^{0}_{ip} - e^{0}_{iq}\right| \leq 0.001\,\mathrm{C/m^2}$ and $\left|e^{int}_{ip} - e^{int}_{iq}\right| \leq 0.001\,\mathrm{C/m^2}$.

2.5 Computation of the Piezoelectric Tensor d_{iq} for Selected MOFs

Piezoelectric calculations for computing d for four MOFs (NAHSQU01, VAFYOQ, BALFAV01, and EHOHOY) in this paper are performed using the ab initio periodic code CRYSTAL17[19] based on the atom-centered Gaussian basis set. Triple zeta basis sets with polarization functions for all atoms[20–24] and Grimme D3 dispersions corrections were used.[15] For Mo in VAFYOQ and BALFAV01 MOFs, extra polarization functions were included.[?, ?] We

adopted DFT functional at the generalized gradient approximation (GGA) level of theory i.e., B3LYP.[25,26] First, we performed a full relaxation (cell parameters and atomic positions) of the MOFs taken from experimentally synthesized structures reported in the CSD database. Using the optimized structure as starting geometry, the full piezoelectric (e, d) and elastic, compliance (c, s) tensors were calculated. For this, we used a numerical approach based on the geometry optimization of the atomic positions in the strained lattice configurations. Piezoelectric constant e is evaluated using the Berry phase approach in the modern theory of polarization as a first derivative of the Berry phase with respect to strain.[27–31] This is done in CRYSTAL17 by applying finite strains ($\pm 1.5\%$) to the lattice and computing the finite differences of polarization at strained configurations. The elastic or compliance constants c or s are expressed as second derivatives of energy with respect to pairs of strains. In CRYSTAL17, the first derivative is computed analytically, while the second derivative is evaluated as numerical finite differences between strained structures described in the work by Erba et al.[32] The other piezoelectric constant d is evaluated from the relation with e and s: $d_{ip} = e_{iq} s_{qp}$.

3 Experimental Methods

3.1 Chemicals and Reagents

Molybdenum trioxide (MoO_3, 99.99%, Aldrich) and 2,2′-Bipyridine (2,2′-bipy, > 99%, Thermoscientific) were used without further purification.

3.2 Synthesis of VAFYOQ

MoO_3(2,2'-bipy)(VAFYOQ) was synthesized according to a literature with slight adjustments.[33] Briefly, a mixture of 0.2117 g of MoO_3, 0.1562 g of 2,2'-bipyridine and 18 mL of DI water was placed in a 40 mL autoclave. The autoclave was then heated at 160 °C for 96 h. After cooling down to room temperature, the products were collected by filtration. Colourless

rods of MoO_3(2,2'-bipy) were manually separated from a mixture of MoO_3(2,2'-bipy) and colourless octahedra Mo_3O_9(2,2'-bipy)$_2$.

3.3 Apparatus and Measurements

Powder X-ray diffraction (PXRD) was performed with a Bruker D8 Advanced diffractometer with a Cu Kα source ($\lambda = 1.5418$ Å). The measured 2θ range was 3° to 70°. Thermogravimetric analysis (TGA) was carried out using a Mettler Toledo 1600 TGA/SDTA851. The measurements were performed in air with a gas flow of 100 mL/min. A pre-treatment at 30 °C for 10 min was employed and then the temperature increased by 10 °C/min up to 800 °C.

The SHG microscopy setup is a Bergam II system by ThorLabs. The light source is a mode-locked Ti:sapphire laser (Vitesse-2W, Coherent) emitting pulses, with a pulse duration of approximately 75 fs to 80 fs, at a wavelength of 800 nm, a repetition rate of 80 MHz, and an average power of approximately 160 mW power. The incident laser light passes subsequently through a laser power controller, an automated half wave plate to control the plane of polarization and galvanometric mirrors. The latter cause a scanning pattern in the x-y plane of the sample via two high-speed vibrating mirrors driven by galvanometer motors, resulting in a scanning type of microscopy. The laser beam is then focused using an objective lens (20x/0.45na) onto a small area on the surface of the sample, the transmitted light passes through a condenser (MBL11300; by Achromat), a polarizer and a bandpass filter centered on 405 nm with 15 nm FWHM bandwidth to ensure that only SHG light reaches the photon-counting photon multiplier tubes (PMTs).

Due to temperature-induced changes in the SHG microscope's focal position, a series of images along the z-axis were acquired at each temperature. These image stacks were then batch processed using ImageJ to obtain the SHG intensity. SHG intensity was quantified using ImageJ's built-in "Measure" function, which calculates the average pixel intensity for each image. For temperature-dependent analysis of the SHG-intensity, the SHG intensity corresponding to the image within each Z-stack with the highest intensity was extracted and

used.

4 BEC of Inorganic Cation in the Secondary Building Units vs Group in the Periodic Table

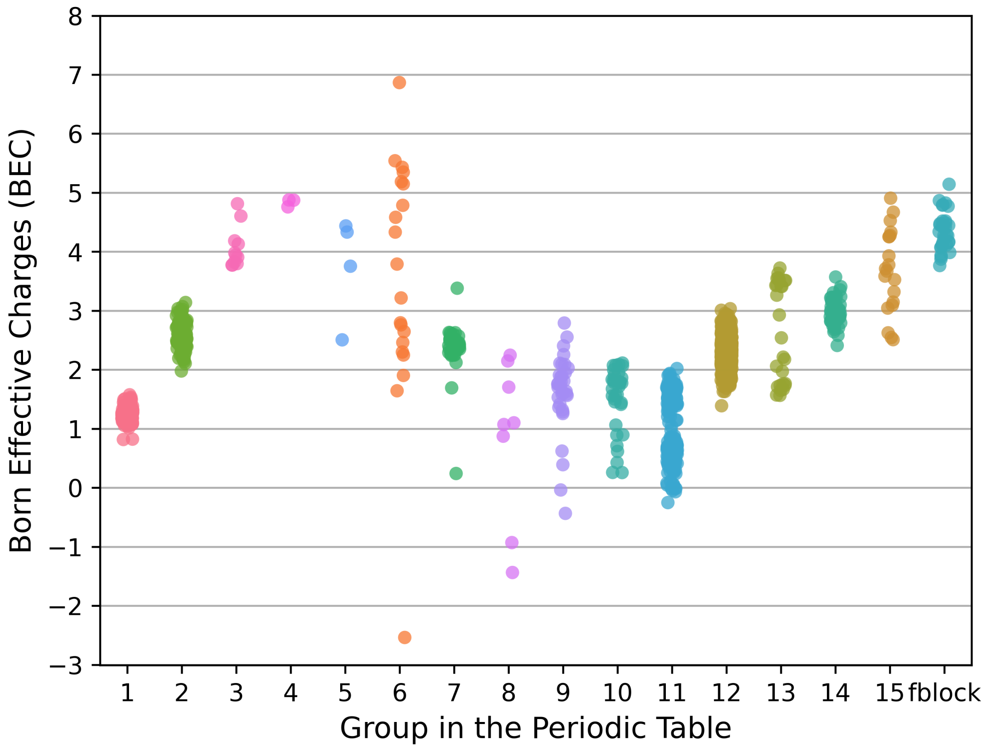

Figure S5: Distribution of BEC values of inorganic cations in MOFs belonging to different groups of the periodic table.

Figure S5 shows the distribution of the BEC values for inorganic cations in MOFs categorized by their respective groups in the periodic table. For Group 1 (alkali metals) which usually have a single oxidation state (OS) of +1, the BEC values are in the range of ∼ OS to OS +0.8, and for Group 2 (alkaline earth metals) with an OS of +2, the BEC values are in the range of ∼ OS to OS+1. For Groups 3 (Sc family) and 4 (Ti family), which also have a single OS of +3 and +4 respectively, the BEC values are higher and in the range of

OS+1 to OS+2. However, the number of MOFs with inorganic cations from Groups 3 and 4 is very limited in this database. Other groups in the transition metals (Groups 5 to 12) have multiple oxidation states; hence we also see multiple values with a wide range for BEC. Group 13 (Al to Tl) generally exhibits oxidation states of +1 or +3; BEC values for this group are in the same range of +1.5 to +3.8. Then Group 14 (Ge to Pb) can have an oxidation state of +2 or +4; while their BEC is in the same range of values. Group 15 elements (Sb, Bi) and f-block elements (La, Ce, Yb) have BEC between +2.5 to +5 and +4 to +5.5 respectively. In general, these findings suggest that BEC values of +4 and higher for inorganic cations in MOFs are observed among elements in Groups 3, 4, 5, 6, 15, and the f-block.

5 Structure Determination of Mo-MOF BALFAV01

5.1 Energy Calculations of Mo-MOF BALFAV01

In the BALFAV01 experimental structure ('2' point group), the bond lengths along y-direction are O1—Mo: 1.918Å and Mo—O2: 1.926Å, resulting in a minimal difference of 0.008Å. Along the x-direction, O1—Mo: 1.917Å and Mo—O2: 1.918Å have equal bond lengths.[34] In the optimized structure, the O1—Mo—O2 bonds exhibit alternating bond lengths forming a long—short—long—short··· pattern (see Figure S6). We computed and compared the DFT energies and structural differences of three structures:

1. Experimental structure in '1' polar point group (no symmetry structure) with unequal O1—Mo and Mo—O2 bond lengths in y-direction.

2. Experimental structure in '2' polar point group with unequal O1—Mo and Mo—O2 bond lengths in y-direction.

3. A non-polar structure in '222' point group with equalized O1—Mo and Mo—O2 bond lengths along the x- and y- directions.

The optimized structure of the '1' polar point group shows significantly pronounced differences in bond length along the x- and y-directions and is shown in Table S1. In case of polar point group structure '2', the difference in bond length is pronounced only along the y-direction. In the non-polar structure, due to symmetry restrictions, the bond lengths O1—Mo and Mo—O2 are equal. When comparing the DFT energies of these 3 structures, the polar structure with '1' point group is found to be energetically favorable, indicating that it represents the global minima. The alternating long–short–long–short pattern is preserved along x- and y-directions in the relaxed structure, suggesting a robust polar distortion.

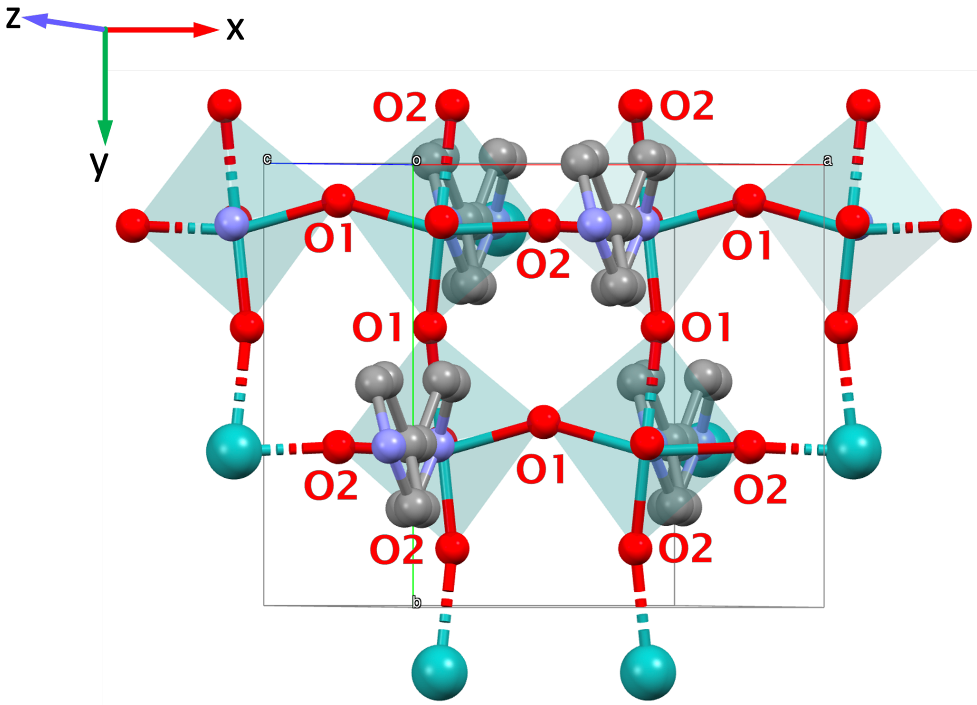

Figure S6: Structure of BALFAV01 (showing the xy plane) and 2-D square grid of O1—Mo—O2 bonds along the x- and y-direction. teal: Molybdenum (Mo), gray: Carbon (C), red: Oxygen (O), white: Hydrogen (H), blue: Nitrogen (N).

Table S1: DFT Energies, Bond length (BL) differences in Mo—O bonds for Polar ('1'), Polar ('2') and Non-Polar ('222') BALFAV01 structures.

Point Group	Polar ('1')	Polar ('2')	Non-Polar ('222')
BL O1—Mo, Mo—O2 along x (Å)	1.803, 2.028	1.880, 1.897	1.923, 1.923
BL difference along x (Å)	**0.225**	**0.017**	**0**
BL O1—Mo, Mo—O2 along y (Å)	1.769, 2.137	1.754, 2.243	1.888, 1.888
BL difference along y (Å)	**0.368**	**0.489**	**0**
DFT Energy (Ha)	-1082.810	-1082.807	-1082.799
Energy ('1')− Energy (Structure) (kJ/mol)	**0**	**-6.536**	**-27.878**

5.2 PXRD and SHG of Mo-MOF VAFYOQ

Due to the needle morphology of VAFYOQ, its preferential orientation significantly influences the relative intensities of peaks in the PXRD pattern. In the crystal structure of VAFYOQ, the O—Mo—O chains are aligned along the z-axis. Correspondingly, the diffraction peaks from crystal planes parallel to the O—Mo—O direction, such as (110), (220), (330), and (440), exhibited the highest intensities compared to other peaks, confirming the direction along the needle length aligns with the c-axis.

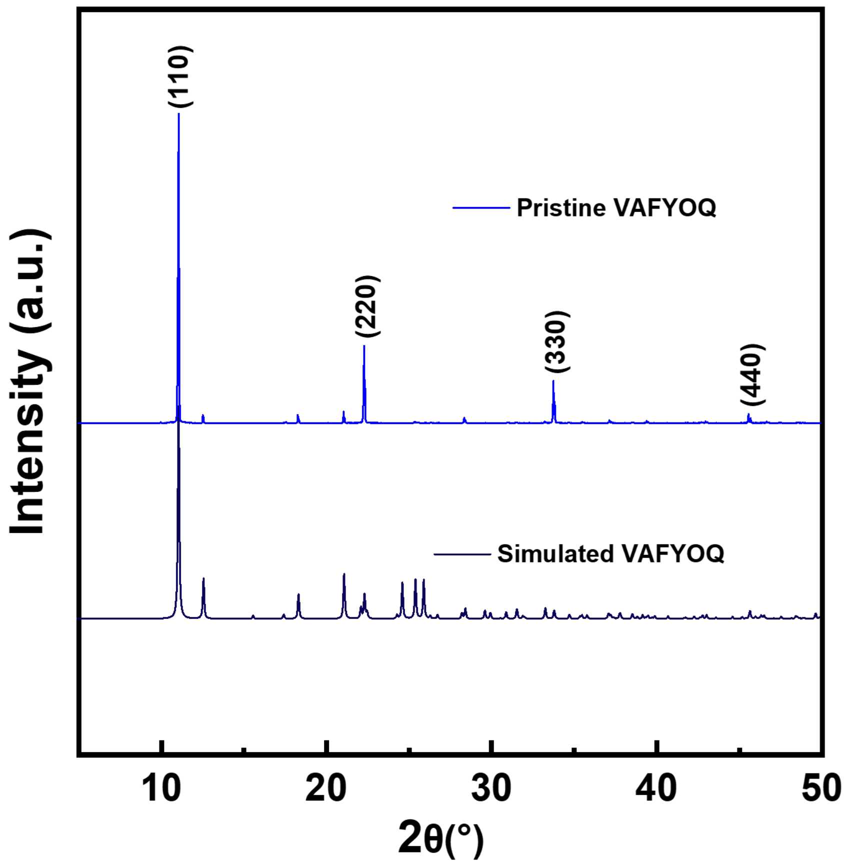

Figure S7: PXRD of VAFYOQ.

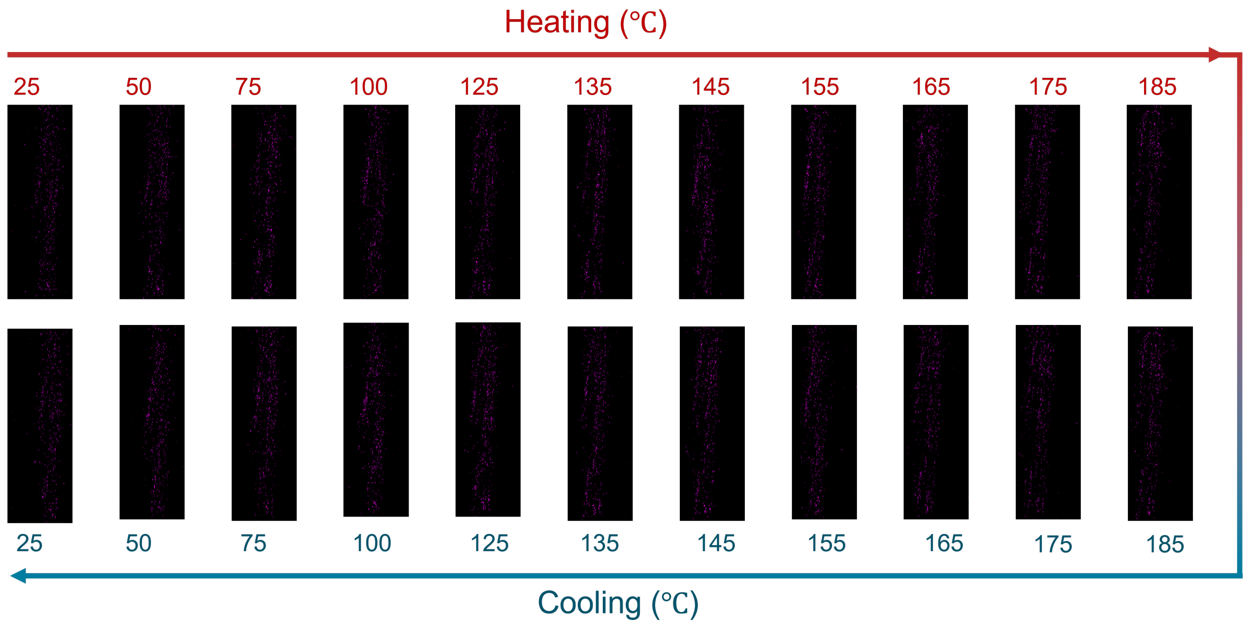

Figure S8: SHG images of VAFYOQ crystal upon heating and cooling when the incident and detected light is polarized is perpendicular to O—Mo—O.

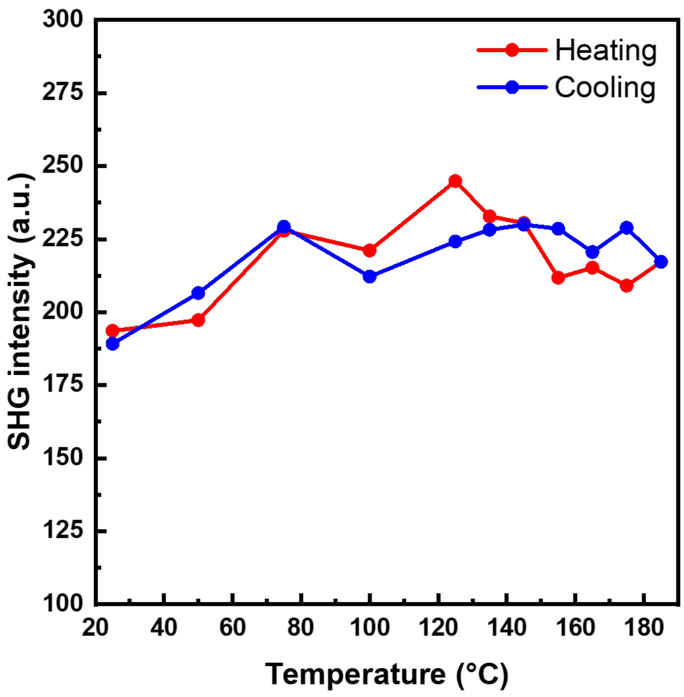

Figure S9: SHG intensity change of VAFYOQ crystal upon heating and cooling when the incident and detected light is polarized perpendicularly to O—Mo—O.

6 Piezoelectric Tensor e_{iq} and Born Effective Charges Z^* of the Inorganic Cations for Selected MOFs

6.1 YECPEE

$$\text{Piezoelectric tensor } e_{iq} = \begin{bmatrix} -0.002 & -0.038 & -0.059 & 0. & -0.062 & 0. \\ 0. & 0. & 0. & 0.169 & 0. & 0.011 \\ -1.234 & 0.594 & -0.124 & 0. & -0.727 & 0. \end{bmatrix}$$

Norm value of the piezoelectric tensor $\left\|e_{iq}\right\|_{max} = 1.556\,\mathrm{C/m^2}$

$$\text{Born effective charge (BEC) of K, } Z_K^* = \begin{bmatrix} 1.209 & 0.392 & -0.011 \\ 0.119 & 1.754 & -0.014 \\ 0.006 & -0.031 & 1.119 \end{bmatrix}$$

Average $Z_K^* = +1.36$

6.2 NAHSQU01

$$\text{Piezoelectric tensor } e_{iq} = \begin{bmatrix} 0.447 & 0.152 & 0.584 & 0. & 0.426 & 0. \\ 0. & 0. & 0. & 0.095 & 0. & 0.032 \\ 0.195 & 0.103 & 0.565 & 0. & 0.607 & 0. \end{bmatrix}$$

Norm value of the piezoelectric tensor $\left\|e_{iq}\right\|_{max} = 1.198\,\mathrm{C/m^2}$

$$\text{Born effective charge (BEC) of Na, } Z^*_{Na} = \begin{bmatrix} 1.045 & 0.044 & 0.079 \\ 0.032 & 1.391 & 0.208 \\ 0.025 & 0.080 & 1.173 \end{bmatrix}$$

$$\text{Average } Z^*_{Na} = +1.20$$

6.3 AGALUR

$$\text{Piezoelectric tensor } e_{iq} = \begin{bmatrix} 0. & 0. & 0. & 0.150 & 0. & 0. \\ 0. & 0. & 0. & 0. & 0.944 & 0. \\ 0. & -0.000 & 0. & 0. & 0. & -0.004 \end{bmatrix}$$

$$\text{Norm value of the piezoelectric tensor } \left\|e_{iq}\right\|_{max} = 0.944\,\text{C/m}^2$$

$$\text{Born effective charge (BEC) of Mg, } Z^*_{Mg} = \begin{bmatrix} 2.036 & 0.053 & -0.035 \\ -0.121 & 2.497 & -0.021 \\ -0.062 & 0.016 & 2.048 \end{bmatrix}$$

$$\text{Average } Z^*_{Mg} = +2.19$$

6.4 VAFYOQ

$$\text{Piezoelectric tensor } e_{iq} = \begin{bmatrix} -0.426 & -0.143 & 0.156 & 0. & 0.019 & 0. \\ 0. & 0. & 0. & -0.034 & 0. & -0.343 \\ 0.371 & 0.295 & -1.382 & 0. & -0.216 & 0. \end{bmatrix}$$

$$\text{Norm value of the piezoelectric tensor } \left\|e_{iq}\right\|_{max} = 1.506\,\text{C/m}^2$$

$$\text{Born effective charge (BEC) of Mo, } Z^*_{Mo} = \begin{bmatrix} 3.866 & 0.164 & -0.639 \\ 0.031 & 3.708 & 0.270 \\ -0.423 & -0.782 & 8.715 \end{bmatrix}$$

Average $Z^*_{Mo} = +5.43$

6.5 BALFAV01

$$\text{Piezoelectric tensor } e_{iq} = \begin{bmatrix} 2.361 & -0.735 & -0.324 & -0.017 & 0.010 & 0.042 \\ -0.697 & 1.445 & -0.130 & -0.109 & 0.008 & 0.067 \\ 0.011 & -0.063 & -0.012 & 0.005 & 0.005 & -0.008 \end{bmatrix}$$

Norm value of the piezoelectric tensor $\left\|e_{iq}\right\|_{max} = 2.763\,\mathrm{C/m^2}$

$$\text{Born effective charge (BEC) of Mo, } Z^*_{Mo} = \begin{bmatrix} 8.994 & -0.101 & 1.287 \\ -0.955 & 7.059 & 0.215 \\ 0.020 & 0.301 & 4.353 \end{bmatrix}$$

Average $Z^*_{Mo} = +6.86$

6.6 EHOHOY

$$\text{Piezoelectric tensor } e_{iq} = \begin{bmatrix} -1.076 & -0.258 & -0.073 & 0. & -0.227 & 0. \\ 0. & 0. & 0. & 0.294 & 0. & 0.121 \\ -0.173 & 0.225 & 0.316 & 0. & -0.548 & 0. \end{bmatrix}$$

Norm value of the piezoelectric tensor $\left\|e_{iq}\right\|_{max} = 1.159\,\mathrm{C/m^2}$

$$\text{Born effective charge (BEC) of Sb, } Z^*_{Sb} = \begin{bmatrix} 4.024 & 0.184 & 0.628 \\ 0.127 & 3.313 & 0.400 \\ 0.533 & 0.443 & 4.448 \end{bmatrix}$$

$$\text{Born effective charge (BEC) of P, } Z^*_{P} = \begin{bmatrix} 3.731 & 0.205 & 0.332 \\ -0.768 & 3.896 & 0.064 \\ -0.102 & -0.902 & 3.188 \end{bmatrix}$$

$$\text{Average } Z^*_{Sb} = +3.93$$

$$\text{Average } Z^*_{P} = +3.60$$

6.7 GAXQAZ

$$\text{Piezoelectric tensor } e_{iq} = \begin{bmatrix} 0.527 & -0.087 & 0.415 & 0. & 0.403 & 0. \\ 0. & 0. & 0. & 0.037 & 0. & 0. \\ -0.518 & 0.0651 & -0.371 & 0. & -0.405 & 0. \end{bmatrix}$$

$$\text{Norm value of the piezoelectric tensor } \left\| e_{iq} \right\|_{max} = 1.092\,\mathrm{C/m^2}$$

$$\text{Born effective charge (BEC) of Zn, } Z^*_{Zn} = \begin{bmatrix} 2.242 & 0.065 & 0.153 \\ 0.035 & 1.920 & 0.090 \\ 0.199 & 0.133 & 2.237 \end{bmatrix}$$

$$\text{Average } Z^*_{Zn} = +2.13$$

6.8 UVAROZ

$$\text{Piezoelectric tensor } e_{iq} = \begin{bmatrix} -0.505 & -0.361 & -0.724 & 0. & -0.056 & 0. \\ 0. & 0. & 0. & -0.013 & -0. & 0.041 \\ -0.288 & -0.139 & -0.353 & -0. & 0.017 & -0. \end{bmatrix}$$

$$\text{Norm value of the piezoelectric tensor } \left\|e_{iq}\right\|_{max} = 1.066\,\mathrm{C/m^2}$$

$$\text{Born effective charge (BEC) of Zn, } Z^*_{Zn} = \begin{bmatrix} 3.372 & 0.063 & -0.051 \\ -0.028 & 2.011 & -0.002 \\ -0.510 & -0.125 & 2.730 \end{bmatrix}$$

$$\text{Average } Z^*_{Zn} = +2.71$$

7 Dielectric tensor ε_{ij} for Selected MOFs

7.1 YECPEE

$$\text{Dielectric Tensor (Clamped-ion Contribution), } \varepsilon^0_{ij} = \begin{bmatrix} 2.947 & 0 & -0.063 \\ 0 & 3.027 & 0 \\ -0.063 & 0 & 2.39 \end{bmatrix}$$

$$\text{Dielectric Tensor (Relaxed-ion Contribution), } \varepsilon^{ion}_{ij} = \begin{bmatrix} 2.892 & 0 & 0.834 \\ 0 & 4.607 & -0.001 \\ 0.834 & -0.001 & 15.471 \end{bmatrix}$$

7.2 NAHSQU01

$$\text{Dielectric Tensor (Clamped-ion Contribution), } \varepsilon_{ij}^{0} = \begin{bmatrix} 2.515 & 0 & 0.600 \\ 0 & 2.864 & 0 \\ 0.600 & 0 & 2.696 \end{bmatrix}$$

$$\text{Dielectric Tensor (Relaxed-ion Contribution), } \varepsilon_{ij}^{ion} = \begin{bmatrix} 3.813 & 0 & 2.087 \\ 0 & 2.591 & 0 \\ 2.087 & 0 & 5.102 \end{bmatrix}$$

7.3 AGALUR

$$\text{Dielectric Tensor (Clamped-ion Contribution), } \varepsilon_{ij}^{0} = \begin{bmatrix} 2.545 & 0 & 0 \\ 0 & 2.580 & 0 \\ 0 & 0 & 2.244 \end{bmatrix}$$

$$\text{Dielectric Tensor (Relaxed-ion Contribution), } \varepsilon_{ij}^{ion} = \begin{bmatrix} 2.808 & 0 & 0 \\ 0 & 8.326 & 0 \\ 0 & 0 & 1.820 \end{bmatrix}$$

7.4 VAFYOQ

$$\text{Dielectric Tensor (Clamped-ion Contribution)}, \varepsilon_{ij}^{0} = \begin{bmatrix} 3.541 & 0 & -0.029 \\ 0 & 3.916 & 0 \\ -0.029 & 0 & 3.198 \end{bmatrix}$$

$$\text{Dielectric Tensor (Relaxed-ion Contribution)}, \varepsilon_{ij}^{ion} = \begin{bmatrix} 0.642 & 0 & -0.793 \\ 0 & 0.921 & 0 \\ -0.793 & 0 & 12.652 \end{bmatrix}$$

7.5 BALFAV01

$$\text{Dielectric Tensor (Clamped-ion Contribution)}, \varepsilon_{ij}^{0} = \begin{bmatrix} 3.709 & 0 & 0.045 \\ 0 & 3.462 & 0 \\ 0.045 & 0 & 3.558 \end{bmatrix}$$

$$\text{Dielectric Tensor (Relaxed-ion Contribution)}, \varepsilon_{ij}^{ion} = \begin{bmatrix} 49.869 & 0 & -14.437 \\ 0 & 2.087 & 0 \\ -14.437 & 0 & 4.939 \end{bmatrix}$$

7.6 EHOHOY

$$\text{Dielectric Tensor (Clamped-ion Contribution)}, \varepsilon_{ij}^{0} = \begin{bmatrix} 3.587 & 0 & 0.069 \\ 0 & 3.764 & 0 \\ 0.069 & 0 & 3.555 \end{bmatrix}$$

$$\text{Dielectric Tensor (Relaxed-ion Contribution)}, \varepsilon_{ij}^{ion} = \begin{bmatrix} 7.360 & 0 & 3.879 \\ 0 & 6.332 & 0 \\ 3.879 & 0 & 9.599 \end{bmatrix}$$

7.7 GAXQAZ

$$\text{Dielectric Tensor (Clamped-ion Contribution)}, \varepsilon_{ij}^{0} = \begin{bmatrix} 2.911 & 0 & -0.025 \\ 0 & 2.478 & 0 \\ -0.025 & 0 & 2.507 \end{bmatrix}$$

$$\text{Dielectric Tensor (Relaxed-ion Contribution)}, \varepsilon_{ij}^{ion} = \begin{bmatrix} 4.751 & 0 & -3.355 \\ 0 & 0.586 & 0 \\ -3.355 & 0 & 3.868 \end{bmatrix}$$

7.8 UVAROZ

$$\text{Dielectric Tensor (Clamped-ion Contribution)}, \varepsilon_{ij}^{0} = \begin{bmatrix} 2.896 & 0 & -0.227 \\ 0 & 2.711 & 0 \\ -0.227 & 0 & 3.030 \end{bmatrix}$$

$$\text{Dielectric Tensor (Relaxed-ion Contribution)}, \varepsilon_{ij}^{ion} = \begin{bmatrix} 2.328 & 0 & 0.111 \\ 0 & 0.803 & 0 \\ 0.111 & 0 & 0.754 \end{bmatrix}$$

8 Piezoelectric Tensor d_{iq}, e_{iq} and Compliance Tensor s_{pq} for Selected MOFs

The piezoelectric tensor d_{iq} and compliance tensor s_{pq} is calculated for four MOFs among the top 8 high performing MOFs with $\left\|e_{iq}\right\|_{max} \geq 1.0\,\mathrm{C/m^2}$. The four MOFs are, an alkali metal-based MOF (NAHSQU01), two Mo–MOFs (BALFAV01 and VAFYOQ) and Sb–based MOF (EHOHOY). The piezoelectric tensor e_{iq} is also obtained along with d_{iq} and s_{pq} in these calculations. These calculations are done using CRYSTAL17 and the starting structures are taken from experimental structures deposited in the CSD database. The piezoelectric tensor e_{iq} was previously calculated in this work for these four MOFs in high-throughput DFPT calculations using VASP. For the VASP calculations, we started from computationally optimized structures from the QMOF database. We observed slight differences in the values of piezoelectric tensor e_{iq} between VASP and CRYSTAL17 generated for the same MOF. The differences between the VASP and CRYSTAL17 computed values of the piezoelectric norm are $0.32\,\mathrm{C/m^2}$, $0.08\,\mathrm{C/m^2}$, $0.11\,\mathrm{C/m^2}$, $0.03\,\mathrm{C/m^2}$ for NAHSQU01, VAFYOQ, BALFAV01, and EHOHOY respectively. In the literature, discrepancies of about 15-20% are commonly reported between different DFT based methods for piezoelectric

constants in inorganic materials.[35] In this study, the difference between piezoelectric norm values computed using VASP and CRYSTAL17 is approximately 25% for NAHSQU01, and between 2–5% for VAFYOQ, BALFAV01, and EHOHOY. These variations can be attributed to several factors, including the use of different computational frameworks (plane wave vs Gaussian basis set),theoretical approaches for evaluating the piezoelectric tensor (DFPT vs finite geometry method), exchange–correlation functionals (PBE vs B3LYP) and starting structure (computationally optimized vs experimental geometry). Overall, this level of agreement is within the acceptable range for DFT based calculations, and the observed differences do not effect the general trends or the design principles proposed in this work.

8.1 NAHSQU01

$$\text{Piezoelectric tensor in pC/N}, d_{iq} = \begin{bmatrix} 1.918 & 0.762 & 1.427 & 0 & 9.079 & 0 \\ 0 & 0 & 0 & 4.729 & 0 & 0.987 \\ -6.247 & -1.326 & 11.051 & 0 & 3.605 & 0 \end{bmatrix}$$

$$\text{Norm value of the piezoelectric tensor } \left\| d_{iq} \right\|_{max} = 13.731\,\text{pC/N}$$

$$\text{Piezoelectric tensor in C/m}^2, e_{iq} = \begin{bmatrix} 0.171 & 0.15 & 0.399 & 0 & 0.273 & 0 \\ 0 & 0 & 0 & 0.087 & 0 & 0.031 \\ 0.094 & 0.066 & 0.609 & 0 & 0.333 & 0 \end{bmatrix}$$

$$\text{Norm value of the piezoelectric tensor } \left\| e_{iq} \right\|_{max} = 0.875\,\text{C/m}^2$$

$$\text{Compliance tensor in TPa}^{-1}, s_{pq} = \begin{bmatrix} 36.429 & 10.621 & 28.529 & 0 & 5.759 & 0 \\ 10.621 & 102.72 & 23.659 & 0 & 1.946 & 0 \\ 28.529 & 23.659 & 65.7 & 0 & 25.5670 & \\ 0 & 0 & 0 & 17.363 & 0 & 5.239 \\ 5.759 & 1.946 & 25.567 & 0 & 24.645 & 0 \\ 0 & 0 & 0 & 5.239 & 0 & 6.04 \end{bmatrix}$$

8.2 VAFYOQ

$$\text{Piezoelectric tensor in pC/N}, d_{iq} = \begin{bmatrix} 16.575 & 3.401 & -18.174 & 0 & -7.904 & 0 \\ 0 & 0 & 0 & 6.901 & 0 & 17.947 \\ -21.607 & -18.177 & 50.372 & 0 & 39.879 & 0 \end{bmatrix}$$

$$\text{Norm value of the piezoelectric tensor } \left\|d_{iq}\right\|_{max} = 74.102\,\text{pC/N}$$

$$\text{Piezoelectric tensor in C/m}^2, e_{iq} = \begin{bmatrix} 0.460 & 0.173 & -0.443 & 0 & 0.101 & 0 \\ 0 & 0 & 0 & 0.087 & 0 & 0.256 \\ -0.32 & -0.226 & 1.423 & 0 & -0.066 & 0 \end{bmatrix}$$

$$\text{Norm value of the piezoelectric tensor } \left\|e_{iq}\right\|_{max} = 1.585\,\text{C/m}^2$$

$$\text{Compliance tensor in TPa}^{-1}, s_{pq} = \begin{bmatrix} 39.392 & -25.309 & -11.378 & 0 & -21.933 & 0 \\ -25.309 & 62.980 & -8.221 & 0 & 4.798 & 0 \\ -11.378 & -8.221 & 32.967 & 0 & 30.734 & 0 \\ 0 & 0 & 0 & 159.868 & 0 & -27.235 \\ -21.933 & 4.798 & 30.734 & 0 & 148.250 & 0 \\ 0 & 0 & 0 & -27.235 & 0 & 79.427 \end{bmatrix}$$

8.3 BALFAV01

$$\text{Piezoelectric tensor in pC/N}, d_{iq} = \begin{bmatrix} 86.248 & -55.205 & -15.242 & -41.997 & -1.639 & -7.991 \\ -46.489 & 45.031 & 3.675 & 23.280 & 10.108 & 10.545 \\ 1.478 & -1.665 & -0.362 & -0.441 & 6.278 & 0.109 \end{bmatrix}$$

Norm value of the piezoelectric tensor $\|d_{iq}\|_{max} = 131.449\,\text{pC/N}$

$$\text{Piezoelectric tensor in C/m}^2, e_{iq} = \begin{bmatrix} 2.125 & -0.836 & -0.268 & 0.020 & 0.014 & 0.006 \\ -0.780 & 1.370 & -0.146 & -0.107 & 0 & 0.073 \\ 0.015 & -0.065 & -0.020 & 0.011 & 0.013 & -0.003 \end{bmatrix}$$

Norm value of the piezoelectric tensor $\|e_{iq}\|_{max} = 2.646\,\text{C/m}^2$

$$\text{Compliance tensor in TPa}^{-1}, s_{pq} = \begin{bmatrix} 46.366 & 29.621 & 20.444 & -1.871 & 0.383 & 0 \\ 29.621 & 65.281 & 13.700 & -9.727 & 0.165 & -1.707 \\ 20.444 & 13.700 & 110.892 & -9.898 & 1.293 & -0.205 \\ -1.871 & -9.727 & -9.898 & 12.063 & -0.049 & 0.011 \\ 0.383 & 0.165 & 1.293 & -0.049 & 2.150 & -1.440 \\ 0 & -1.707 & -0.205 & 0.011 & -1.440 & 18.364 \end{bmatrix}$$

8.4 EHOHOY

$$\text{Piezoelectric tensor in pC/N}, d_{iq} = \begin{bmatrix} -12.600 & -0.999 & 3.391 & 0 & -6.484 & 0 \\ 0 & 0 & 0 & 11.787 & 0 & 42.089 \\ -0.356 & 5.505 & 8.532 & 0 & -38.384 & 0 \end{bmatrix}$$

$$\text{Norm value of the piezoelectric tensor } \left\|d_{iq}\right\|_{max} = 43.708\,\text{pC/N}$$

$$\text{Piezoelectric tensor in C/m}^2, e_{iq} = \begin{bmatrix} -1.117 & -0.36 & -0.011 & 0 & -0.163 & 0 \\ 0 & 0 & 0 & 0.294 & 0 & 0.139 \\ 0.025 & 0.36 & 0.337 & 0 & -0.636 & 0 \end{bmatrix}$$

$$\text{Norm value of the piezoelectric tensor } \left\|e_{iq}\right\|_{max} = 1.187\,\text{C/m}^2$$

$$\text{Compliance tensor in TPa}^{-1}, s_{pq} = \begin{bmatrix} 87.741 & 22.283 & 11.517 & 0 & 4.302 & 0 \\ 22.283 & 93.012 & 19.429 & 0 & 8.076 & 0 \\ 11.517 & 19.429 & 58.656 & 0 & 6.941 & 0 \\ 0 & 0 & 0 & 38.540 & 0 & -3.811 \\ 4.302 & 8.076 & 6.941 & 0 & 19.219 & 0 \\ 0 & 0 & 0 & -3.811 & 0 & 4.360 \end{bmatrix}$$

References

(1) IEEE Standard on Piezoelectricity. *ANSI/IEEE Std 176-1987* **1988**,

(2) Nye, J. F. *Physical properties of crystals: their representation by tensors and matrices*, 1st ed.; Clarendon Press ; Oxford University Press: Oxford [Oxfordshire] : New York, 1984.

(3) Sághi-Szabó, G.; Cohen, R. E.; Krakauer, H. First-Principles Study of Piezoelectricity in PbTiO3. *Physical Review Letters* **1998**, *80*, 4321–4324.

(4) Dal Corso, A.; Posternak, M.; Resta, R.; Baldereschi, A. Ab initio study of piezoelectricity and spontaneous polarization in ZnO. *Physical Review B* **1994**, *50*, 10715–10721.

(5) Catti, M.; Noel, Y.; Dovesi, R. Full piezoelectric tensors of wurtzite and zinc blende ZnO and ZnS by first-principles calculations. *Journal of Physics and Chemistry of Solids* **2003**, *64*, 2183–2190.

(6) Ghosez, P.; Michenaud, J.; Gonze, X. Dynamical atomic charges: The case of ABO3 compounds. *Physical Review B - Condensed Matter and Materials Physics* **1998**, *58*, 6224–6240.

(7) Rosen, A. S.; Iyer, S. M.; Ray, D.; Yao, Z.; Aspuru-Guzik, A.; Gagliardi, L.; Notestein, J. M.; Snurr, R. Q. Machine learning the quantum-chemical properties of metal–organic frameworks for accelerated materials discovery. *Matter* **2021**, *4*, 1578–1597.

(8) Moghadam, P. Z.; Li, A.; Wiggin, S. B.; Tao, A.; Maloney, A. G.; Wood, P. A.; Ward, S. C.; Fairen-Jimenez, D. Development of a Cambridge Structural Database Subset: A Collection of Metal-Organic Frameworks for Past, Present, and Future. *Chemistry of Materials* **2017**, *29*, 2618–2625.

(9) Baroni, S.; Giannozzi, P.; Testa, A. Green's-function approach to linear response in solids. *Physical Review Letters* **1987**, *58*, 1861–1864.

(10) Baroni, S.; de Gironcoli, S.; Dal Corso, A.; Giannozzi, P. Phonons and related crystal properties from density-functional perturbation theory. *Reviews of Modern Physics* **2001**, *73*, 515–562.

(11) Gonze, X. Adiabatic density-functional perturbation theory. *Physical Review A* **1995**, *52*, 1096–1114.

(12) Kresse, G.; Furthmüller, J. Efficient iterative schemes for ab initio total-energy calculations using a plane-wave basis set. *Physical Review B* **1996**, *54*, 11169–11186.

(13) Kresse, G.; Joubert, D. From ultrasoft pseudopotentials to the projector augmented-wave method. *Physical Review B* **1999**, *59*, 1758–1775.

(14) Perdew, J. P.; Burke, K.; Ernzerhof, M. Generalized Gradient Approximation Made Simple. *Physical Review Letters* **1996**, *77*, 3865–3868.

(15) Grimme, S.; Antony, J.; Ehrlich, S.; Krieg, H. A consistent and accurate ab initio parametrization of density functional dispersion correction (DFT-D) for the 94 elements H-Pu. *The Journal of Chemical Physics* **2010**, *132*, 154104.

(16) Grimme, S.; Ehrlich, S.; Goerigk, L. Effect of the damping function in dispersion corrected density functional theory. *Journal of Computational Chemistry* **2011**, *32*, 1456–1465.

(17) Blöchl, P. E. Projector augmented-wave method. *Physical Review B* **1994**, *50*, 17953–17979.

(18) Mula, S.; Donà, L.; Civalleri, B.; van der Veen, M. A. Structure–Property Relationship of Piezoelectric Properties in Zeolitic Imidazolate Frameworks: A Computational Study. *ACS Applied Materials & Interfaces* **2022**, *14*, 50803–50814, Publisher: American Chemical Society.

(19) Dovesi, R.; Erba, A.; Orlando, R.; Zicovich-Wilson, C. M.; Civalleri, B.; Maschio, L.; Rérat, M.; Casassa, S.; Baima, J.; Salustro, S.; Kirtman, B. Quantum-mechanical condensed matter simulations with CRYSTAL. *WIREs Computational Molecular Science* **2018**, *8*, e1360.

(20) Francl, M. M.; Pietro, W. J.; Hehre, W. J.; Binkley, J. S.; Gordon, M. S.; DeFrees, D. J.; Pople, J. A. Self-consistent molecular orbital methods. XXIII. A polarization-type basis set for second-row elements. *The Journal of Chemical Physics* **1982**, *77*, 3654–3665.

(21) Krishnan, R.; Binkley, J. S.; Seeger, R.; Pople, J. A. Self-consistent molecular orbital methods. XX. A basis set for correlated wave functions. *The Journal of Chemical Physics* **1980**, *72*, 650–654.

(22) McLean, A. D.; Chandler, G. S. Contracted Gaussian basis sets for molecular calculations. I. Second row atoms, Z=11-18. *The Journal of Chemical Physics* **1980**, *72*, 5639–5648.

(23) Heyd, J.; Peralta, J. E.; Scuseria, G. E.; Martin, R. L. Energy band gaps and lattice parameters evaluated with the Heyd-Scuseria-Ernzerhof screened hybrid functional. *Journal of Chemical Physics* **2005**, *123*, 174101.

(24) Pritchard, B. P.; Altarawy, D.; Didier, B.; Gibson, T. D.; Windus, T. L. New Basis Set Exchange: An Open, Up-to-Date Resource for the Molecular Sciences Community. *Journal of Chemical Information and Modeling* **2019**, *59*, 4814–4820.

(25) Raghavachari, K. Perspective on "Density functional thermochemistry. III. The role of exact exchange". *Theoretical Chemistry Accounts: Theory, Computation, and Modeling (Theoretica Chimica Acta)* **2000**, *103*, 361–363.

(26) Adamo, C.; Barone, V. Toward reliable density functional methods without adjustable parameters: The PBE0 model. *J. Chem. Phys* **1999**, *110*, 6158.

(27) King-Smith, R. D.; Vanderbilt, D. Theory of polarization of crystalline solids. *Physical Review B* **1993**, *47*, 1651–1654.

(28) Resta, R. Macroscopic polarization in crystalline dielectrics: the geometric phase approach. *Reviews of Modern Physics* **1994**, *66*, 899–915.

(29) Vanderbilt, D. Berry-phase theory of proper piezoelectric response. *Journal of Physics and Chemistry of Solids* **2000**, *61*, 147–151, arXiv: cond-mat/9903137 ISBN: 0022-3697.

(30) Dall'Olio, S.; Dovesi, R.; Resta, R. Spontaneous polarization as a Berry phase of the Hartree-Fock wave function: The case of KNbO3. *Physical Review B* **1997**, *56*, 10105–10114.

(31) Noel, Y.; Zicovich-Wilson, C. M.; Civalleri, B.; D'Arco, P.; Dovesi, R. Polarization properties of ZnO and BeO: An ab initio study through the Berry phase and Wannier functions approaches. *Physical Review B* **2001**, *65*, 014111.

(32) Erba, A.; Caglioti, D.; Zicovich-Wilson, C. M.; Dovesi, R. Nuclear-relaxed elastic and piezoelectric constants of materials: Computational aspects of two quantum-mechanical approaches. *Journal of Computational Chemistry* **2017**, *38*, 257–264.

(33) Zapf, P. J.; Haushalter, R. C.; Zubieta, J. Hydrothermal Synthesis and Structural Characterization of a Series of One-Dimensional Organic/Inorganic Hybrid Materials of the [$(MoO_3)_n$ (2,2'-bipy)$_m$] Family: [MoO_3(2,2'-bipy)], [Mo_2O_6(2,2'-bipy)], and [Mo_3O_9(2,2'-bipy)$_2$]. *Chemistry of Materials* **1997**, *9*, 2019–2024.

(34) Cui, C.-P.; Dai, J.-C.; Du, W.-X.; Fu, Z.-Y.; Hu, S.-M.; Wu, L.-M.; Wu, X.-T. Synthesis, structure and fluorescence of a 3-D polymer {(Mo4O12)(4,4-bipy)2}n. *Polyhedron* **2002**, *21*, 175–179.

(35) de Jong, M.; Chen, W.; Geerlings, H.; Asta, M.; Persson, K. A. A database to enable discovery and design of piezoelectric materials. *Scientific Data* **2015**, *2*, 150053.
